# Modeling left ventricular diastolic dysfunction: classification and key indicators

**DOI:** 10.1186/1742-4682-8-14

**Published:** 2011-05-09

**Authors:** Chuan Luo, Deepa Ramachandran, David L Ware, Tony S Ma, John W Clark

**Affiliations:** 1 Dept. Electrical and Computer Engineering, Rice University, Houston, TX 77005, USA; 2 Div. Cardiology, University of Texas Medical Branch, Galveston, TX 77555, USA; 3 Div. Cardiology, VA Medical Center, Houston, Texas 77030, USA; 4 Baylor College of Medicine, One Baylor Plaza, Houston, Texas 77030, USA

## Abstract

**Background:**

Mathematical modeling can be employed to overcome the practical difficulty of isolating the mechanisms responsible for clinical heart failure in the setting of normal left ventricular ejection fraction (HFNEF). In a human cardiovascular respiratory system (H-CRS) model we introduce three cases of left ventricular diastolic dysfunction (LVDD): (1) impaired left ventricular active relaxation (IR-type); (2) increased passive stiffness (restrictive or R-type); and (3) the combination of both (pseudo-normal or PN-type), to produce HFNEF. The effects of increasing systolic contractility are also considered. Model results showing ensuing heart failure and mechanisms involved are reported.

**Methods:**

We employ our previously described H-CRS model with modified pulmonary compliances to better mimic normal pulmonary blood distribution. IR-type is modeled by changing the activation function of the left ventricle (LV), and R-type by increasing diastolic stiffness of the LV wall and septum. A 5^th^-order Cash-Karp Runge-Kutta numerical integration method solves the model differential equations.

**Results:**

IR-type and R-type decrease LV stroke volume, cardiac output, ejection fraction (EF), and mean systemic arterial pressure. Heart rate, pulmonary pressures, pulmonary volumes, and pulmonary and systemic arterial-venous O_2 _and CO_2 _differences increase. IR-type decreases, but R-type increases the mitral E/A ratio. PN-type produces the well-described, pseudo-normal mitral inflow pattern. All three types of LVDD reduce right ventricular (RV) and LV EF, but the latter remains normal or near normal. Simulations show reduced EF is partly restored by an accompanying increase in systolic stiffness, a compensatory mechanism that may lead clinicians to miss the presence of HF if they only consider LVEF and other indices of LV function. Simulations using the H-CRS model indicate that changes in RV function might well be diagnostic. This study also highlights the importance of septal mechanics in LVDD.

**Conclusion:**

The model demonstrates that abnormal LV diastolic performance alone can result in decreased LV and RV systolic performance, not previously appreciated, and contribute to the clinical syndrome of HF. Furthermore, alterations of RV diastolic performance are present and may be a hallmark of LV diastolic parameter changes that can be used for better clinical recognition of LV diastolic heart disease.

## Background

Frequently, heart failure symptoms occur in the presence of a normal left ventricular ejection fraction (HFNEF), however, some do not regard "diastolic heart failure" as synonymous with HFNEF, because diastolic abnormalities alone may not fully explain the phenomenon [1,2]. The cause, proper assessment, and very name of this syndrome have been debated. This controversy requires broadened investigation to improve treatments for the disease. Certainly the interaction of all possible causes makes it very difficult in practice to determine the extent to which any one might be responsible. Zile et al. [3] have reported that patients with clinical diastolic heart failure have demonstrable abnormalities of left ventricular (LV) active relaxation and passive stiffness. This modeling paper tries to demonstrate that: (1) the reverse is true; that by selectively altering the active relaxation and passive stiffness parameters of the septum and LV free wall, clinical parameters of different diastolic HF are produced by model simulation; (2) by combining alterations of active relaxation and passive stiffness parameters, a phenotype is produced which parallels the pseudonormal diastolic HF; (3) LVEF is normal when increased LV systolic contractility is considered; and (4) by analyzing this modeling exercise, new diagnostic clinical parameters of diastolic heart disease are classified and proposed. This study aims to shed light on one of the many causes of HFNEF, that of left ventricular diastolic dysfunction (LVDD).

Mathematical models help by predicting the hemodynamic, pulmonary, and neural responses to isolated changes in each parameter under investigation. Our group has developed a detailed human cardiovascular respiratory system model (H-CRS) [4-8] that reproduces normal and abnormal hemodynamic, respiratory, and neural physiology. Although the model is comparatively complex [8,9] it provides a very comprehensive and integrated explanation of cardiovascular and respiratory events, such as thigh-cuff and carotid occlusion [6], the Valsalva maneuver [4], the pumping action of the interventricular septum [8], and atrioventricular and interventricular interactions in cardiac tamponade [11]. The model has been fit to pooled systemic and pulmonary arterial impedance data [12,13] and its echocardiographic flow and pressure measurements agree well with those of normal humans [7]. Comparing model predictions with echocardiographic findings and key indices in patients with HFNEF might help to explain which, or to what extent each of the possible abnormalities is responsible for the disease.

## Methods

### H-CRS Model

The present iteration of the H-CRS model [4-7,14] includes a few updates from the one described in [7] including: a) a new description of the distribution of the pulmonary blood volume according to data from Ohno et al. [15], wherein pulmonary compliance values more accurately match normal pulmonary blood distribution (see Appendix B); and b) an altered pericardial model as detailed in [11]. All model differential equations associated with the current version of the model are listed in Appendix A. This closed-loop, composite model is a system of ordinary differential equations with state variables such as chamber pressures, chamber volumes, and transvalvular flows. Ventricular free walls and septum are driven by independent activation functions, therefore producing time-varying RV, LV and septal elastance. Important model parameters are given in Appendix B.

The instantaneous pressure (mmHg) within either left or right ventricular free wall (LVF or RVF) volume (V_LVF _and V_RVF_) (ml) is the weighted sum of pressure during diastole and systole [6]:(1)

where(2)

and(3)

Since both free wall pressures (P_LVF_, P_RVF_) are transmural (differential) pressures with reference to pericardial pressure (P_PERI_), the absolute chamber pressures P_LV _and P_RV _(relative to atmosphere) are equivalent to the respective free wall transmural pressure plus P_PERI_.

The trans-septal pressure difference (mmHg) is:(4)

Septal volume (V_SPT_), or the volume that is traversed by the septum, is calculated from the difference in the two free wall pressures, and is the weighted sum of diastolic and systolic contributions.

If P_SPT _≥ 0,(5)

If P_SPT _< 0,(6)

Septal volume is then the weighted sum of septal volume in systole and diastole:(7)

**Figure 1:**
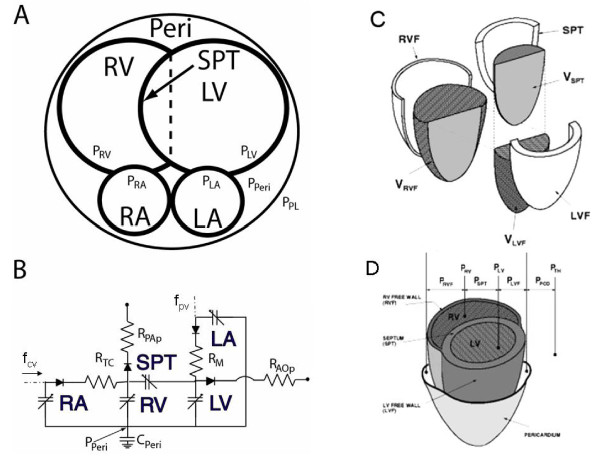
**Coupled Pump Model of Heart**. Panels 1A,C-D show coupled "pump model" of the human heart, with its chamber volumes and pressures. Panel 1B shows hydraulic equivalent circuit model, with diode-resistance pairs representing the pressure-dependent behavior of the tricuspid and mitral (inlet) valves R_TC _and R_M_; and the pulmonic and aortic (outlet) valves R_PAp _and R_AOp_. Time-varying compliances of the right atrium (RA), right ventricle (RV), left atrium (LA), left ventricle (LV), and septum (SPT) are included. The compliance of the pericardium (C_PERI_) is time-invariant.

Given the model storage element volumes (V_LVF_, V_RVF _and V_SPT_), the corresponding transmural pressures for the free walls and septum can be calculated. Cardiac chamber volumes are defined in Figure 1A, and 1C-D. Total left ventricular volume (V_LV_) and right ventricular volume (V_RV_) are defined as:(8)

In these equations e_x_(t) is the dimensionless weight or "activation function," denoting myocardial activation as between 0 and 1, where x = LVF, RVF, or SPT. Ventricular mechanics is described by separate mechanical and temporal behavior - mechanical behavior by static free wall pressure-volume characteristics and temporal behavior by e_x_(t) functions. Thus, the equations for {P_LV,ES_, P_RV,ES_} and {P_LV,ED_, P_RV,ED_} describe the static ESPVR and EDPVR relationships for the ventricular free walls. Here {V_LVF,d_, V_RVF,d_, V_SPT,d_} and {V_LVF,0_, V_RVF,0_, V_SPT,0_} are the zero-pressure volumes for the systolic and diastolic pressure relationships, respectively, whereas the elastance terms {E_LVF,ES_, E_RVF,ES_, E_SPT,ES_} characterize the slopes of linear end-systolic P-V relationships of the LVF and RVF and septum (mmHg/ml). The function α(F_con_) is a dimensionless neural control factor; {λ_LV_, λ_RV_, λ_SPT_} are stiffness parameters associated with the passive diastolic pressure relationships (ml^-1^); and {P_LVF,0_, P_RVF,0_, P_SPT,0_} are the nominal diastolic pressures for the LVF, RVF and septum.

We model both free walls and septum as undergoing independent activation; thus each has its own activation function e_x_(t). Baseline or "control" simulations are those which model normal physiology, and for these we used the activation functions that reproduced normal ventricular pressure tracings.

The solution procedure begins with estimated values for V_SPT_, V_LV_, and V_RV_, and we iterate as follows:

Step 1: V_LVF _= V_LV _- V_SPT_

V_RVF _= V_RV _+ V_SPT_

Step 2: Calculate P_LVF _and P_RVF _(Eqn. 1) using the free wall components of Eqns. 2-3.

Step 3: Calculate P_SPT _according to Eqn. 4.

Step 4: Repeatedly solve for V_SPT _(Eqn. 7) until the septal components of Eqns. 5-6 converge (about 12 iterations).

Step 5: Compute the chamber volumes V_LV _and V_RV _(Eqn. 8), which serve as state variables.

Elastance functions representing the time-varying stiffness of the storage compartments are evaluated using the equations given below:(9)

In addition to increasing ventricular contractility, the baroreflex decreases vagal and increases sympathetic efferent discharge frequency to the sinus node and the peripheral vasculature, increasing heart rate and vasomotor tone.

### Modeling LVDD

LVDD refers to an abnormality in left ventricle's ability to fill during diastole. Diastole is that portion of the cardiac cycle concerned with active relaxation of the ventricle followed by mitral valve opening, ventricular filling, late atrial contraction and mitral valve closure, which signals the end of the diastolic period. Conventional Doppler echocardiographic techniques for measuring mitral flow velocity have yielded flow patterns characteristic of at least two distinct types of LVDD (impaired relaxation (IR) and restrictive (R)). Our modeling approach suggests that a third type of Doppler flow pattern called the pseudo-normal (PN) pattern can be represented simply as a weighted combination of the two basic flow patterns (IR and R). Analysis of these different flow patterns have contributed to a preliminary classification of LVDD.

In an attempt to model the more global consequences of LVDD rather than just its effect on left heart mechanics, we compare the hemodynamic waveforms generated by our H-CRS model of normal physiology, with those generated by the same model, but with modified left ventricular mechanics. In this comparison, only parameters concerned with LV mechanics are changed to produce mitral flow patterns consistent with the three patterns observed in IR-type, R-type, and PN-type LVDD. Thus, three sets of parameter changes were used to generate three different LV models, which were subsequently inserted into the LV compartment of the H-CRS model for testing. Hemodynamic waveforms generated by each of these LV mechanics characterizations were subsequently compared with normal human control waveforms and those generated by the other LV models. The specific modeling mechanisms used to characterize the different LVDD mitral valve patterns are discussed below. The LVDD models are chosen such that they produce typical mitral flow patterns characteristic of the LVDD type, and such that the severity of LVDD produced increases in order IR-type→R-type→PN-type.

#### IR-type

The generic activation function e_x_(t) associated with Eqns. (1) and (7) above is characterized by a sum of Gaussian functions  with amplitude A, width B, and offset C. It varies between 0 and 1, increasing during systole and falling during diastole. End-systole occurs at the peak of or just after the peak of the e_x_(t) curve, and its declining limb drives the dynamics of LV ventricular pressure during isovolumic relaxation. Ideally this phase is nearly complete when the AV (mitral and tricuspid) valves open. *Impaired relaxation *of the LV is a condition that prolongs isovolumic relaxation time resulting in delayed mitral valve opening, elevated LV filling pressure, and reduced mitral flow and end-diastolic volume. To better characterize this flow pattern we increased parameter B in the last Gaussian term for the LVF and septal activation functions from 40 (control) to 350 ms (Table 1). This required adjusting the last two Gaussian terms to normalize e_x_(t) to 1. As a result, LVF relaxation is delayed, the LV end-diastolic pressure-volume relation (EDPVR) has an increased slope and shifts upward and to the left relative to its control curve, and e_x_(t) has a non-zero positive offset at end-diastole. Thus, modeling IR-type requires modifying specific parameters associated with the activation functions of the LVF and septum.

**Table 1:** Gaussian Coefficients for Ventricular and Septal Activation Functions

Gaussian	1	2	3	4	5	6	7
**A**	0.282	0.075	0.384	0.205	0.37	0.516 (0.37)	0.15 (0.249)

**B (sec)**	0.043	0.03	0.05	0.04	0.08	0.06	0.04 (0.35)

**C (sec)**	0.11	0.165	0.22	0.3	0.35	0.395	0.405

Gaussian coefficients for the ventricular and septal activation functions , where x = {LVF, RVF, SPT}. The e_x_(t) coefficient values for the free walls and septum are the same in control. However, with impaired relaxation, the values in the 6^th ^and 7^th ^terms (in parentheses) are used for both the LVF and the septum.

#### R-type

The *restrictive *flow velocity pattern seen in LVDD reflects increased passive wall stiffness of the LVF and septum. In this pattern, the EDPVR has an increased slope relative to its control, end-diastolic volume is reduced and end-diastolic pressure is increased substantially which strongly reduces mitral flow. The effects of increased LV passive wall stiffness were simulated by increasing the diastolic stiffness parameter λ_LV _from 0.025 to 0.05/ml and λ_SPT _from 0.05 to 0.1/ml. Thus, modeling R-type LVDD modifies specific parameters associated with the passive stiffness of the LVF and septum, in mimicking R-type flow pattern in LVDD. R-type LVDD was also modeled with a normal septum (R_NSPT_-type) for analysis of septal contribution.

#### PN-type

As mentioned previously, the *pseudo-normal *flow velocity pattern is viewed as a combined IR + R pattern where one may use a variety of weighting factors in forming the combination. We have chosen to represent the IR and R patterns so that they have nearly equal effect in terms of changes observed in the LV pressure-volume relationship, and have combined them equally to represent the PN case. Specifically, we changed the last Gaussian term B to 350 ms, λ_LV _to 0.05/ml, and λ_SPT _to 0.1/ml. All other H-CRS model parameters remained at control values.

#### Systolic Contractility

Given the report of Kawaguchi et al. [1] that systolic contractility increases to maintain left ventricular stroke volume (LVSV) and cardiac output (CO) within the setting of LVDD, we repeated these simulations after first increasing the gain of the LV end-systolic pressure-volume relationship (E_LV,ES _and E_SPT,ES_) by 60%. If the diastolic stiffness of the muscle fibers of the wall increase with no stimulation, then with stimulation of the very same fibers and subsequent development of normal active tension, logically there should be some increase in total developed tension (active + passive) compared with the control case. Consequently, an increase in the gain of the end-systolic pressure-volume relationships (E_LV,ES _and E_SPT,ES_) should be evident.

This increase in "systolic contractility" is considered intrinsically myogenic in nature (i.e., heterometric autoregulation of cardiac output on the basis of fiber length as in the Frank-Starling mechanism) and is not due to reflex sympathetic augmentation in myocardial contractility. This later form of contractility control is present in the H-CRS model, but it is a separate mechanism that affects the ESPVR via the function α(F_con_) in Eqn. 2 above.

For all cases, we further examined how each condition affects the systemic, pulmonary, and cerebral circulations. Unless otherwise specified, the pleural pressure was held at -5 mmHg in all simulations to eliminate respiratory variations in inlet valve flows and thus better focus on hemodynamic events.

### Computational Aspects

The model consists of 93 nonlinear ordinary differential equations plus 6 embedded diffusion equations that describe the distributed gas exchange compartments of the lung, tissue, and brain. A 5^th^-order Cash-Karp Runge-Kutta [16] numerical integration method solves the differential equations on an IBM compatible PC. Simulating 50 seconds takes about 1 hour to compute using a Pentium 4 2.4G machine with 512 MB DDR RAM.

## Results

### Normal Physiology

**Figure 2:**
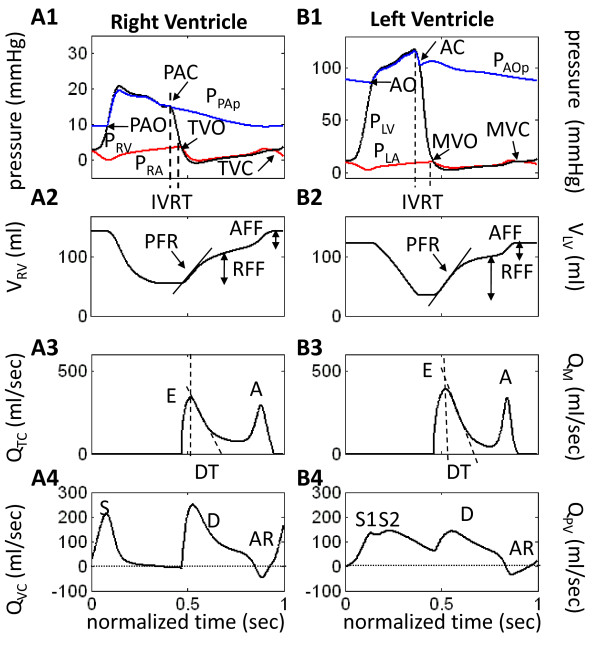
**Model Waveforms for Right and Left Ventricles in Control Case**. Model-generated pressure, volume and flow waveforms for the *normal patient *(control case). PFR = peak filling rate (slope of drawn line); RFF = rapid filling fraction; AFF = atrial filling fraction; IVRT = isovolumic relaxation time; DT = E-wave deceleration time; (P)AO/C = (pulmonary) aortic valve opens/closes; MVO/C = mitral valve opens/closes; TVO/C = tricuspid valve opens/closes.

**Figure 3:**
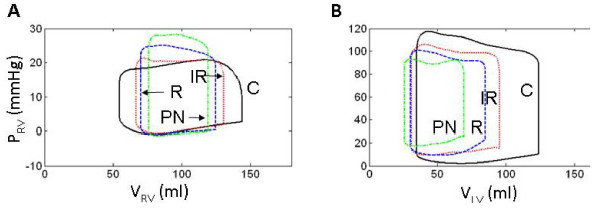
**Comparison of Model Ventricular Pressure-Volume Loops**. Comparing modeled ventricular function curves of normal physiology (C, solid black line) with LVDD due to impaired LV wall relaxation (IR, dotted red line), increased LV wall stiffness (R, dashed blue line), and combined impaired relaxation and increased wall stiffness (PN, dash-dot magenta line). Panels A and B show RV and LV chamber pressures and volumes, respectively. All simulations here are with normal systolic contractility. LVDD types: IR (impaired relaxation); R (resistive) and PN (pseudo-normal) patterns (discussed later).

Model-generated tracings of normal cardiac function are shown in Figure 2 for the right (Panels A1-A4) and left ventricles (Panels B1-B4). These are considered control waveforms for comparison with simulations of diastolic dysfunction. Of particular note are the tricuspid (Q_TC_) and mitral (Q_M_) flow waveforms shown in Figure 2A3 and 2B3, respectively. These waveforms have an early (E wave) and a late (A wave) component during diastolic ventricular filling. Normally, the E/A ratio is 1 - 1.5 and the trans-mitral deceleration time (DT; Figure 2B3) during rapid filling (E wave) is 170 - 230 ms [7]. The central venous (Q_VC_) and distal pulmonary venous (Q_PV_) flow waveforms are shown in Figure 2A4 and 2B4, respectively. These waveforms consist of systolic (S), diastolic (D) and atrial reversal (AR) flow components. The normal systolic pulmonary venous S wave is split into early and late components (S1 and S2; Figure 2B4). Table 2 lists the indices for both right and left ventricular performance and the mean values of systemic circulatory variables, blood gas tensions, and A-V gas differences in the brain and extra-cranial tissues. Figure 3 (solid black line labeled C for control) depicts the normal instantaneous RV and LV pressure-volume relationships. The other loops and curves of the three modeled LVDD types are discussed below.

**Table 2:** Model Values for Key Indices and Variables in Control and LVDD Cases

Parameter	Control		Impaired Relaxation		Restrictive Filling		Pseudo-Normalization	
**e**_**LV,SPT**_**(t)**	normal	normal	altered	altered	normal	normal	altered	altered

**Contractility**	normal	increased	normal	increased	normal	increased	normal	increased

**λ**_**DAP**_**=λ**_**DAM **_**(1/ml)**	0.05	0.05	0.05	0.05	0.1	0.1	0.1	0.1

**λ**_**LV **_**(1/ml)**	0.025	0.025	0.025	0.025	0.05	0.05	0.05	0.05

**E**_**LV,ES **_**(mmHg/ml)**	3.5	5.6	3.5	5.6	3.5	5.6	3.5	5.6

**LVEDP (mmHg)**	10.5	9.5	16.0	17.0	23.3	21.4	25.0	25.0

**MSAP (mmHg) mHg)(mmHg)**	96.6	98.4	91.5	91.3	89.0	90.6	84.9	85.3

**CVP (mmHg)**	2.4	2.6	1.4	1.4	0.2	0.6	-0.6	-0.6

**HR (beats/min)**	55.2	53.9	60.2	60.6	63.8	61.4	68.2	68.1

**CO (l/min)**	4.9	5.2	3.9	3.9	3.5	3.9	3.0	3.0

**LVSV (ml)**	89.4	96.0	64.7	63.9	55.3	62.7	43.5	44.1

**LVEF**	0.72	1.4	0.68	0.76	0.65	0.76	0.63	0.72

**LVET (sec)**	0.28	0.31	0.26	0.27	0.29	0.31	0.27	0.28

**RVSV (ml)**	89.1	95.8	64.5	63.7	54.5	61.9	43.3	43.7

**RVEF**	0.62	0.81	0.49	0.48	0.44	0.47	0.37	0.36

**RVET (sec)**	0.38	0.37	0.33	0.33	0.27	0.30	0.22	0.22

**P**_**AO2 **_**(mmHg)**	105.0	104.3	106.5	106.6	108.1	106.2	108.9	108.5

**P**_**ACO2 **_**(mmHg)**	39.3	39.6	38.0	37.7	37.2	37.5	36.3	36.3

**P**_**TO2 **_**(mmHg)**	42.5	43.4	37.2	36.9	33.5	35.8	29.5	29.5

**P**_**TCO2 **_**(mmHg)**	45.4	45.2	46.8	46.9	47.8	47.2	48.4	48.4

**P**_**BO2 **_**(mmHg)**	37.4	37.4	37.2	37.2	36.9	37.2	36.2	36.3

**P**_**BCO2 **_**(mmHg)**	45.3	45.2	45.8	45.9	46.2	46.1	46.2	46.2

**A-V O2 (T)**	4.4%	4.4%	5.9%	6.0%	7.1%	6.2%	9.0%	8.9%

**A-V CO2 (T)**	-1.9%	-1.9%	-2.4%	-2.4%	-2.7%	-2.5%	-3.1%	-3.1%

**A-V O2 (B)**	5.1%	5.1%	5.5%	5.5%	5.9%	5.6%	6.2%	6.2%

**A-V CO2 (B)**	-0.4%	-0.4%	-0.8%	-0.8%	-0.9%	-0.8%	-1.1%	-1.1%

**F**_**HRv**_	0.54	0.54	0.51	0.51	0.50	0.51	0.47	0.39

**F**_**HRs**_	0.28	0.27	0.33	0.33	0.35	0.34	0.39	0.39

**F**_**con**_	0.40	0.38	0.45	0.46	0.49	0.47	0.53	0.53

**F**_**vaso**_	0.54	0.51	0.62	0.62	0.64	0.62	0.70	0.69

**F**_**b**_	0.41	0.41	0.39	0.39	0.38	0.38	0.36	0.36

**F**_**c**_	0.17	0.17	0.16	0.16	0.15	0.16	0.14	0.14

**F**_**cc**_	0.53	0.53	0.54	0.54	0.55	0.55	0.55	0.55

Values calculated for several indices and variables associated with the ventricles, systemic circulation and gas transport. These values are displayed for control conditions (normal heart), and for the three possible forms of LVDD (impaired active relaxation (IR) alone, increased passive stiffness (R) alone, and combined impaired relaxation and increased stiffness (PN)) without (E_LV,ES _= 3.5) and with (E_LV,ES _= 5.6) increased systolic contractility. All are averaged over one respiratory cycle. F_HRv_, F_HRs_, F_con_, F_vaso _are mean baroreceptor frequencies affecting heart rate (vagal and sympathetic components), contractility, and vasomotor tone. P_AO2_, P_TO2 _and P_BO2 _are arterial, systemic venous, and jugular venous partial O_2 _pressures; likewise P_ACO2_, P_TCO2 _and P_BCO2 _are partial CO_2 _pressures.

### Impaired Active Relaxation with Normal Systolic Contractility

**Figure 4:**
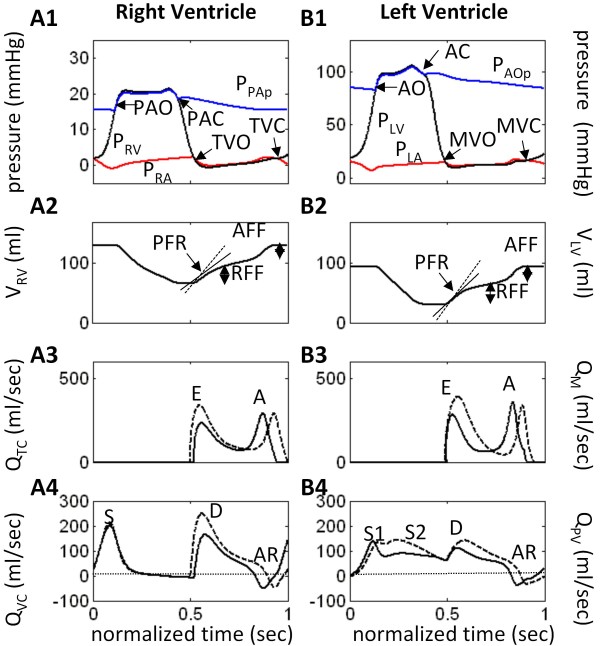
**Model Waveforms for Impaired Relaxation Case**. Model-generated pressure, volume and flow waveforms for the *impaired relaxation *(IR) case. Dashed line plots are the control case for comparison. Abbreviations are as in Figure 2. See text for details.

The P-V loops (Figure 3) show a decrease in LV and RV stroke volume. Cardiac output and mean arterial and central venous pressures decrease (Table 2). Diastolic LV pressure exceeds control throughout diastole in the IR-type case (Figure 3B), elevating LV diastolic and left atrial (LA) pressures (compare Figure 2B1 and Figure 4B1). Pulmonary capillary pressure (P_PC_) increases from 8.5 to 14.0 mmHg, and pulmonary blood volume (V_PC_) by 12.2%, indicating pulmonary congestion (Table 3). Figure 4 reveals even greater detail. The salient features of IR-type are:

**Table 3:** Model Values for Key Pulmonary Indices

Parameter	Control	Impaired Relaxation		Restrictive Filling		Pseudo- normalization	
**λ**_**SPT **_**(1/ml)**	0.05	0.05	0.05	0.1	0.1	0.1	0.1

**λ**_**LV **_**(1/ml)**	0.025	0.025	0.025	0.05	0.05	0.05	0.05

**e**_**LV(and SPT)**_**(t)**	normal	altered	altered	normal	normal	altered	altered

**Contractility**	normal	normal	increased	normal	increased	normal	increased

**E**_**LV,ES **_**(mmHg/ml)**	3.5	3.5	5.6	3.5	5.6	3.5	5.6

**V**_**PA,p **_**(ml)**	20.4	23.6	24.0	26.0	25.0	28.3	28.5

**V**_**PA,d **_**(ml)**	21.1	24.1	24.4	26.3	25.3	28.4	28.6

**V**_**PA **_**(ml)**	201.1	230.4	233.5	251.5	242.1	272.0	273.6

**V**_**PV **_**(ml)**	226.9	267.5	271.4	294.3	282.0	323.1	324.6

**V**_**PC **_**(ml)**	94.9	106.3	107.4	113.9	110.5	122.1	122.5

**P**_**PA,p **_**(mmHg)**	13.2	17.8	18.3	21.3	19.8	24.6	24.9

**P**_**PA,d **_**(mmHg)**	13.0	17.7	18.2	21.2	19.7	24.5	24.8

**P**_**PA **_**(mmHg)**	12.2	17.1	17.6	20.6	19.0	24.0	24.3

**P**_**PV **_**(mmHg)**	7.9	13.4	14.0	17.1	15.4	21.1	21.3

**P**_**PC **_**(mmHg)**	8.5	14.0	14.5	17.6	15.9	21.4	21.7

Mean values for several pressures and volumes associated with the pulmonary circulation.

(a) Reduction in LV end-diastolic volume (EDV) and rates of ejection (Figure 4B2) as shown by the decreased PFR slope (compare with dashed line or control), with a severe reduction in the rapid filling fraction (RFF) relative to control. The atrial filling fraction (AFF) is relatively normal. The normal RV also experiences a reduction in EDV and rates of ejection and early filling (Figure 4A2).

(b) Strong decreases in the early E-wave component of both the mitral and tricuspid flow waveforms (Figure 4A3 and 4B3) reflect the difficulty in ventricular filling. The dashed line waveforms are control, shown for comparison.

(c) There is a pronounced separation of the S1 and S2 components of systolic portion of pulmonary venous flow waveform (Q_PV) _(Figure 4B4), accompanied by a strong reduction in the amplitudes of the S2 component and the D wave. The atrial reversal waveform (AR) is relatively normal in IR-type LVDD. The dashed line waveforms are control, shown for comparison.

The normalized baroreceptor sensory nerve discharge frequency F_b _declines from 0.41 to 0.39 and the normalized aortic chemoreceptor sensory discharge frequency F_c _from 0.17 to 0.16 (Table 2). The increased F_con _(normalized sympathetic efferent discharge frequency controlling contractility) steepens the end-systolic pressure-volume relationship (ESPVR) slope of both ventricles. LV stroke volume decreases from 89.4 to 64.7 ml, and despite a decrease in the LV ejection fraction from 0.72 to 0.68, this number would not be interpreted as systolic failure.

### Restrictive Filling with Normal Systolic Contractility

**Figure 5:**
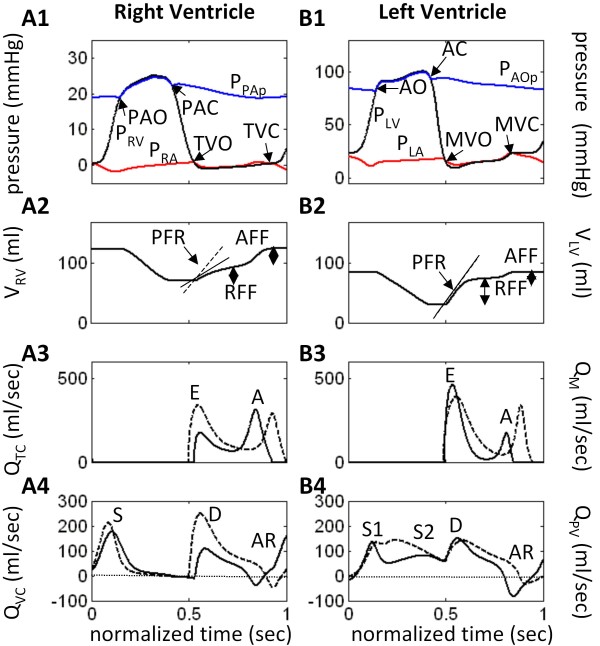
**Model Waveforms for Restrictive Case**. Model-generated pressure, volume and flow waveforms for *restrictive case *(R). Dashed line plots are the control case for comparison. Abbreviations are as in Figure 2. See text for details.

Figure 5 demonstrates the salient characteristics of R-type LVDD:

(a) Reduced EDV (Figure 3) and rates of ejection for both ventricles (Figure 5A2 and 5B2);

(b) Pronounced reduction in RV peak filling rate (PFR) and RFF (Figure 5A2), whereas LV PFR slightly exceeds the control value, but the RFF is reduced relative to control (Figure 5B2). AFF is nearly normal in the RV and strongly reduced in the LV;

(c) With regard to mitral inlet flow (Figure 5B3), the E wave is supra-normal and the A-wave is reduced substantially. This pattern is reversed for the tricuspid flow waveform (Figure 5A3), where the E-wave amplitude is decreased and the A-wave enhanced slightly relative to control (shown by dashed lines);

(d) There is temporal separation of S1 and S2 components of systolic portion of Q_PV _and the amplitude of the S2 component is strongly reduced (Figure 5B4). The diastolic peak of the D waveform is nearly normal, but following the peak it declines faster than the control waveform. The peak of the pulmonary vein AR reversal flow (Figure 5B4) is much enhanced in R-type LVDD. In the central venous flow waveform (Q_VC_; Figure 5A4), the D waveform is strongly reduced and shortened relative to control, the S waveform is only slightly reduced, and the AR reversal flow peak is at control levels.

In the P-V loops of Figure 3, the LV end-diastolic pressure for R-type LVDD is seen to rise relative to control, whereas for the RV they decline slightly relative to control. In contrast, LV systolic pressure declines, but RV systolic pressure is elevated relative to control.

In R-type LVDD, pulmonary pressures and volume increase (Table 3), whereas cardiac output and mean systemic arterial pressure (MSAP) fall by 29% and 7.9%, respectively (Table 2). Calculated LV ejection fraction drops, but only to 0.65.

### Combined Restrictive Filling and Impaired Relaxation with Normal Systolic Contractility

This mechanism causes a marked decrease in right and left ventricular stroke volumes (Figure 3). The LV stroke volume, cardiac output, and mean arterial and central venous pressures decrease by 51.3%, 38.8%, 12.1%, and 125.0%, respectively (Table 2). The baroreceptor reflex responds by reducing vagal discharge frequency (F_HRv_) by 12.2% and increasing sympathetic frequency by 39.3% (F_HRs_). Heart rate increases by 23.6% (Table 2). Once again LV systolic function would not be considered depressed. Its ejection fraction decreases by 12.5%, to 0.63.

**Figure 6:**
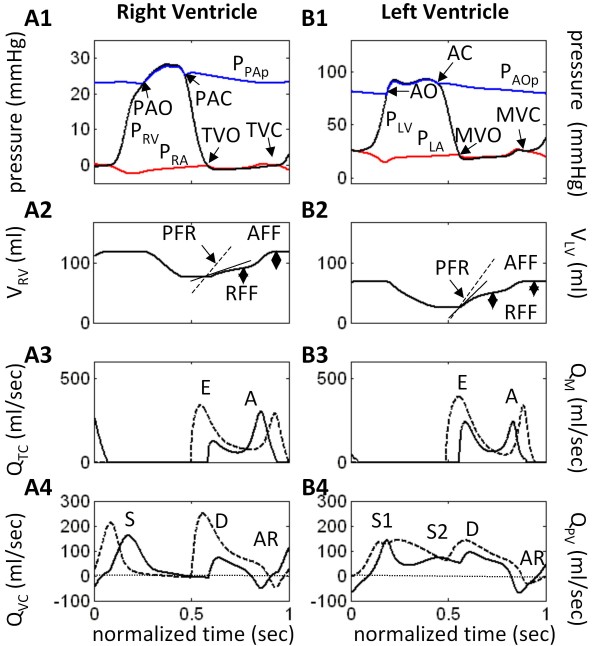
**Model Waveforms for Pseudo-Normal Case**. Model-generated pressure, volume and flow waveforms under *pseudo-normal *conditions (PN = combined IR and R conditions). Dashed line plots are the control case for comparison. Abbreviations are as in Figure 2. See text for details.

Figure 6 shows the detail involved in cardiovascular waveforms associated with PN-type LVDD. The significant features are:

(a) Reduction in EDV in both ventricles to an extent greater than IR or R-type LVDD considered alone (Figure 6A2 and 6B2). Ejection rates and PFRs are decreased substantially in both ventricles, as are RFFs. The LV AFF is strongly reduced, but the RV AFF for the right atrium (RA) is essentially normal;

(b) In the mitral flow waveform, the E and A waves have essentially the same amplitude, whereas the tricuspid flow has an E wave is much smaller than the A wave (Figure 6A3 and 6B2);

(c) There is separation of the S1 and S2 components of systolic portion of the pulmonary venous flow waveform with strong reductions in the S2 component and the diastolic D wave. The AR reversal flow peak is enhanced (Figure 6B4). In the central venous flow waveform, the S wave is reduced in amplitude, the diastolic D wave is attenuated and shortened, and the peak of the AR reversal flow waveform is at control levels (Figure 6A4).

### Septum

**Figure 7:**
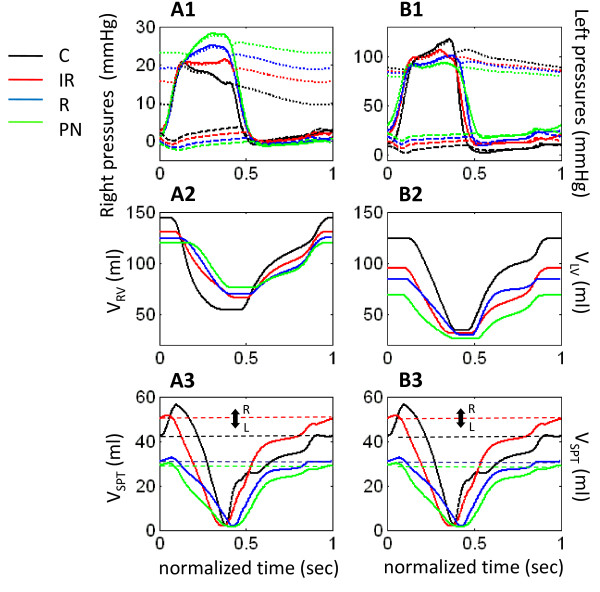
**Time-Aligned Pressures and Volumes**. Time-aligned ventricular and arterial pressures (Panels A1 and A2), chamber volumes (Panels B1 and B2) for the right and left hearts, and septal volume (Panels C1 and C2) in control and different cases of LVDD.

Previous studies from our group show that septal interaction can profoundly affect right heart function [8]. The septum is modeled as an active pump, governed by an activation function, similar to the ventricular free walls. Only such a description for the septum can produce the correct morphology of ventricular pressure tracings seen experimentally as shown by previous work [8,11]. Septal motion can by analyzed by plotting septal volume (V_SPT_), shown in Figure 7A3. Focusing on the control curve (black line) at the beginning of the cycle, with early blood flow into the LV there is an upward movement of the V_SPT _curve which reflects the increased volume of blood in the septum under the influence of the passive left to right pressure gradient across the septum. This initial phase contributes to "priming of the septal pump". As the septum contracts, septal volume decreases indicated by the rapid downward movement of the V_SPT _curve. Thus, increases in septal volume reflect movement of the septum toward the RV, whereas decreases indicate movement of the septum toward the LV (see volumes model in Figure 1A). The septal contractile downstroke ends with closure of the aortic valve, and septal relaxation begins immediately after aortic valve closure. Hence, there is a strong increase in septal volume during the isovolumic relaxation period. This corresponds to rightward movement of the septum which increases septal volume. When the mitral valve opens, the rapid filling phase begins which is marked by a small positive fluctuation in the general exponential filling curve for V_SPT_. The cycle of septal activation and relaxation produces biphasic motion, and as a consequence the septum behaves as a third pump along with the RVF and LVF, and contributes to ventricular performance. Septal priming before contraction initiates RV ejection (Figure 7A2), and RV outflow is maximum just as LV outflow is beginning (see downward slopes in V_RV _and V_LV _in Figure 7A2 and 7B2). This movement simultaneously aids LV filling (Figure 7B2). The following septal contractile leftward thrust provides support to LV ejection (Figure 7B2). V_RV _reaches its minimum point and pulmonary arterial flow ends just before the septum reaches its maximum leftward position at the end of aortic flow (Figure 7A1-A3). In late diastole, the septum returns rightward toward its neutral position (Figure 7A3, black dashed line) as the LV fills and the mitral valve closes (Figure 7B1-B3). The tricuspid valve closes shortly thereafter (Figure 7A2).

In LVDD, the steady-state neutral positions for septal volume changes (marked by dashed lines of corresponding color in Figure 7A3) differ significantly from control. These offsets in the neutral position reflect the different magnitudes of the background left-to-right pressure gradient across the septum in different LVDD states. Septal priming motion is progressively diminished in the order IR→R→PN. In the case of IR-type LVDD, minimal septal priming reduces septal aid in RV ejection, causing pulmonary arterial flow to begin and end later than normal (see downward slope in Figure 7A2). As seen in Figure 7A3, the septum takes longer to reach neutral position so mitral flow lasts longer and its endpoint closer in timing to tricuspid flow (compare end of upward slope in Figure 7A2 and 7B2). The stiffened septum in R-type LVDD does not exhibit priming (Figure 7A3) so there is no elongation of RV ejection and LV filling, and RV and LV outflows are synchronized exactly (downward slopes of Figure 7A2 and 7B2). The septum does not contribute significantly to LV ejection as noted by slower septal leftward stroke and LV volume reaching minimum point before the septum reaches its maximum leftward position (Figure 7B2 and 7B3). As in control, at septal neutral position Q_M _ends while Q_TC _ends shortly thereafter (Figure 7A2-B4). In PN-type LVDD, the septum has little role in determining RV and LV volumes with its minimal and slow movement between ventricles (Figure 7A3). With no septal aid in RV ejection, RV outflow starts much later than aortic flow, and ends later as well (Figure 7A2 and 7B2). The septum also does not influence end-diastolic filling of the RV as in control, and transvalvular flows end at the same time (Figure 7A2 and 7B2).

**Figure 8:**
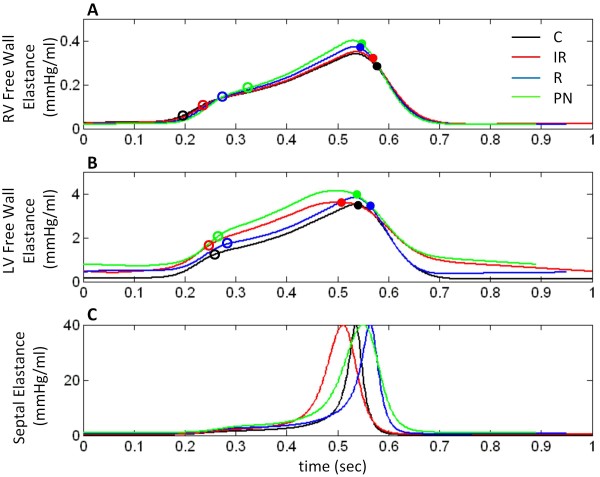
**Ventricular Free Wall and Septal Elastance**. Plots of RV free wall (Panel A), LV free wall (Panel B), and septal (Panel C) elastance. Open circles indicate outlet (pulmonic and aortic) valve opening, closed circles indicate outlet valve closure. Septal elastance bears a sharp peak coincident with RVF and LVF maximum elastance in control (black line). With IR-type (red line), LVF and septal elastance depict abnormal relaxation, and the peaks widen. With R-type (blue line), septal elastance peak is delayed, occurring after free wall elastance peaks, delaying aortic valve closure. With PN-type (green line), plots show signs of both effects with abnormal LVF and septal elastance downstroke, and delayed and widened septal elastance peak. In all LVDD cases, pulmonic valve opening is delayed. See text for details.

Elastance plots provide information about the timing and level of contractility of free walls and septum. Figure 8A-C depicts RVF, LVF, and septal elastance (mmHg/ml) (Eqn. 9). Open circles indicate opening of outlet valves, while solid circles indicate their closure. In the control case (black line), peak elastance occurs simultaneously for all three walls. The aortic valve closes at this peak and the septum, at its maximum leftward position (Figure 7B3) then snaps toward the right showing a sharp drop in septal elastance (Figure 8C) and the pulmonic valve remains open for this final phase of RV ejection (Figure 8A). By comparing the RV and LV ejection periods with the point of occurrence of septal contraction, one can gain a sense of the contribution the septum has to the ejection processes. Specifically, peak elastance coinciding for the LVF and septum (Figure 8B-C) at the point when the septum is leftward in position (Figure 7B3) indicates that its role in LV ejection is maximized as both contract at the same time for efficient ejection. Similarly, RV systole ends only as the septum nears full relaxation (Figure 8A and 8C) indicating septal activity is involved strongly in the RV ejection process.

In IR-type LVDD, the modified activation function for LVF and septum is apparent in elastance curves with a slowed and elevated relaxation phase following peak elastance (Figure 8B-C). Incomplete relaxation maintains the walls in contracted states for a longer time, widening the peaks. The baroreceptor reflex provides a slight positive inotropic effect on RV and LV contractility and elastance (Figure 8A-B) as shown by the F_con _value increasing by 12.5% relative to control. As a result, free wall elastance values exceed control throughout the cardiac cycle. The pulmonic valve opens later than in control (Figure 8A) as seen also in Figure 7A3 due to the loss of septal priming, however the closure time is near control. Thus, RV ejection time is reduced with values shown in Table 2. On the other hand LV ejection time is reduced by premature closure of the aortic valve (Figure 8B).

The LVF elastance curve in R-type LVDD is similar to that for control except for a significant diastolic offset and a higher peak elastance (Figure 8B). RVF elastance however, does not exhibit a diastolic offset, but due to a baroreflex-mediated augmentation of myocardial contractility, the rates of rise and peak elastance are increased (F_con _increases by 22.5% relative to control). A similar sympathetic augmentation applies to the modified (stiffened) LVF elastance; however, the effects of augmentation (other than the increase in peak) are not as evident as in the case of RVF elastance due to masking by neural augmentation (explained below). Both outlet valves open later than in control (Figure 8A-B) resulting in prolongation of both pre-ejection periods (see ejection times in Table 2). Peak septal elastance and thus aortic valve closure occur at a delay from peak LVF elastance (Figure 8C). Unlike in control, the pulmonic valve closes at peak RVF elastance (Figure 8A), well before maximum septal elastance (Figure 8C) and aortic valve closure (Figure 8B). Septal role is diminished for both ventricles: the delay in septal contraction reduces LV ejection support; in the case of the RV, both modes of septal contribution to ejection, initial septal priming and final rightward swing during septal relaxation (Figure 7A3), are lost. RV systolic operation becomes independent of the septum.

In PN-type LVDD, peak LVF elastance decreases but a compensatory increase in F_con _raises this function above control (Figure 8B) (F_con _increases by 32.5% relative to control). This increase in F_con _also increases peak elastance of the RV (Figure 8A). As expected LVF elastance bears effects of impaired relaxation with early peaking, slow and incomplete relaxation, and elevated diastolic elastance exacerbated due to passive stiffness effects (Figure 8B). The septal elastance curve also shows IR effects with a wider peak and slower downward stroke, but a delayed peak resulting from septal stiffness (Figure 8C). Peak elastance values all occur at different times: LVF followed by RVF followed by septum (compare Figure 8A-C). While in IR-type LVDD aortic valve closure precedes pulmonic valve closure and in R-type LVDD the opposite occurs, PN-type LVDD sees a combined effect and outlet valves close at approximately the same time (Figure 8A-B). RV ejection time is severely reduced in comparison to control (see Table 2), due to both pulmonic valve opening delay and early closure (Figure 8A).

**Figure 9:**
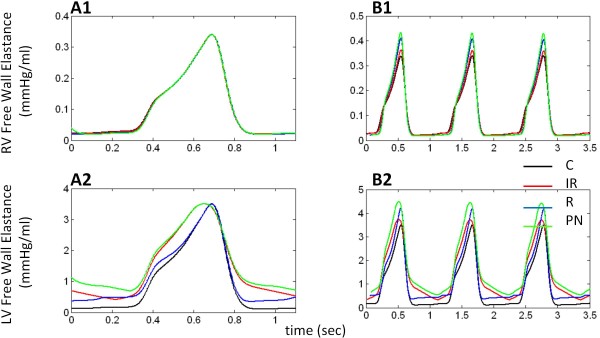
**Model Free Wall Elastance Curves with Loss of Neural Feedback**. RVF and LVF elastance curves with no baroreflex-mediated augmentation of contractility (Panels A1-A2) (model parameter F_con_), and with no heart rate neural control (Panels B1-B2) (model parameters F_HRs _and F_HRv_). With F_con _fixed at mean steady state control levels and no feedback control, RVF elastance does not change, peak elastance remains same as control in all cases. LVF elastance with R-type LVDD exhibits slower rise to peak (unseen in elastance with F_con _in Figure 8). With no FHR_s _and FHR_v_, heart rate is unchanged with LVDD.

To better understand the components affecting elastance, baroreflex-mediated augmentation of myocardial contractility (F_con _parameter) was fixed at mean steady-state control value and RVF and LVF elastance were plotted for a cardiac cycle for the control and LVDD cases (Figure 9A1-A2). This allowed investigation of the hemodynamic consequence of solely LVDD mechanisms. Results show that RVF elastance remains as in control for all LVDD types (Figure 9A1). In IR-type LVDD, LVF elastance peak is wider, has slowed relaxation, and is elevated throughout, except at peak elastance where it matches control and falls below briefly during the downward phase at the start of isovolumic relaxation (red line in Figure 9A2). In R-type LVDD, LVF elastance is elevated above control during diastole, the rise to peak elastance is slower than control, and the peak value matches control (blue line in Figure 9A2). LVF elastance with PN-type LVDD is similar to IR-type, except diastolic elastance is higher and the upstroke slower (green line in Figure 9A2). In all cases, peak elastance does not change (Figure 9A1-A2), unlike what is seen in Figure 8A-B, this feature attributed to neural augmentation of contractility. In addition, R-type LVF elastance is slower on the upstroke (Figure 9A2), this aspect masked when neural augmentation is included making the upstroke appear similar to control. All changes to RVF elastance seen in Figure 8A are also a result of the neural aspect and unrelated to P-V relationships.

Similarly, the reason for heart rate changes with LVDD was evaluated by fixing autonomous neural control of heart rate at mean steady-state control value. RVF and LVF elastance are plotted in Figure 9B1-B2, respectively, for several cardiac cycles. With this feedback missing, heart rate remains unchanged from control in all LVDD types, so any change in heart rate observed in LVDD is solely a result of neural compensation for stroke volume drop.

### Summary of Pressure and Volume Changes

Figure 7 shows that morphology of the pressure and volume waveforms change dramatically from control in the LVDD cases. The disease process is assumed localized to the LV, yet some of the more substantial effects of LVDD are seen in the altered waveforms of the normal right heart. In control, RV pressure slopes downward during ejection under normal pulmonary arterial loading conditions (Figure 7A1), due to the proper operation of the septum which supports LV ejection during this time period. In all of the LVDD cases, the increase in pulmonary arterial afterload and diminished septal contractile motion cause the RV pressure during ejection to change slope in a positive direction. The effect of the LVDD-induced afterloading and decreased septal activity is also seen in the reduced ejection rates in the RV volume curves (Figure 7B1). With a loss of septal contractile motion in LVDD, the LV is not as well-supported and the slope of the P_LV _waveform declines during ejection (Figure 7A2). The volume curves indicate reduced ejection and filling rates and a reduction in stroke volume, hence cardiac output (Figure 7B2). Mean systemic arterial pressure (MSAP) has a tendency to drop, but baroreflex mechanisms compensate to keep systemic arterial load pressure relatively constant. MSAP however does decline slightly from control in each LVDD state (Figure 7B2). Diastolic LV pressure however, changes significantly from control in a positive direction. This strongly affects mitral flow, ventricular filling and ultimately stroke volume. In contrast, diastolic variation in diastolic RV pressure is relatively small and in the negative direction from control (Figure 7A1). Systolic RV pressure varies much more significantly due to increased myocardial contractility.

### Summary of Transvalvular Flow Changes

In the case of the mitral valve, each LVDD state has different effects on the E and A wave components of ventricular filling. Restrictive filling (Figure 5) shortens deceleration time (DT) and increases the E/A ratio (> 1.5), whereas impaired relaxation (Figure 4) slightly prolongs DT and decreases the E/A ratio (< 1). In PN-type (Figure 6) the E and A peaks are nearly equal. The amplitude and duration of the A wave changes considerably relative to control, where in the restrictive case it is small and brief and in IR it has an amplitude and duration comparable to control (slightly increased amplitude; slightly decreased duration). However, in the case of the tricuspid valve, all three LVDD cases yield prolonged deceleration times and abnormal E/A ratios (< 1). The normalized diastolic filling phase is shortened and the amplitudes of the A wave increase slightly relative to control. Thus the E/A ratio of tricuspid flow is more specific than mitral for LVDD, because pseudo-normalization does not occur. In general and depending on the severity of abnormality, tricuspid E-wave flows progressively decrease with LVDD type (IR → R → PN), causing a diminished rapid filling fraction and prolonged deceleration times.

### Summary of Pulmonary and Central Venous Flow Changes

Pulmonary venous flow patterns in simulated LVDD exhibit a strong attenuation in the amplitude of the S2 wave and delay in its peak (Figure 4B4, Figure 5B4, and Figure 6B4). The S1 peak appears early relative to control and is relatively constant amplitude for all LVDD states. The peak of the diastolic D wave varies considerably with LVDD; it is reduced in IR-type and PN-type, but at control levels in R-type. The decay rate of the D wave in restrictive LVDD is markedly increased leading into a very strong AR flow waveform. This strong backflow explains where the blood flow associated with the LA contractile effort went due to the restrictive downstream conditions in the LV chamber (small A wave in the mitral flow waveform (Figure 5B3)). Thus, AR flow peaks are elevated relative to control in R-type and PN-type, but remain at control levels in IR-type. Central venous flow waveforms in LVDD show a decline in peak and a broadening of the S wave with LVDD state, coupled with a strong decline in both peak amplitude and duration of the D wave.

The ratio of D/S flow volume for both the central and pulmonary venous flows can indicate change in inflow patterns. For example, lowering ratios are indicative of lesser diastolic contribution to ventricular inflow. Pulmonary venous flow volume drops from the control value of 0.74 with all LVDD cases except the restrictive case, wherein it increases (Table 4). In central venous flow volume, all LVDD cases show lowered D/S ratios compared to the control value of 1.96. Lowered D/S ratios are indicative of higher diastolic pressures, preventing complete filling of the atria. The higher pulmonary venous D/S ratio in restrictive LVDD is influenced by the limited ventricular pumping action during systole, thereby restricting LA inflow.

**Table 4:** D/S Ratios of Central and Pulmonary Venous Flow Volumes

D/S Ratio	Control	IR	R	PN
**Right**	1.96 (100%)	1.10 (-44%)	0.67 (-66%)	0.30 (-85%)

**Left**	0.74 (100%)	0.57 (-23%)	0.85(+15%)	0.46 (-38%)

Diastolic-to-systolic ratios of central (right) and pulmonary (left) venous flow volumes into the heart. Except for pulmonary flow volume in the R-type LVDD case, the D/S ratio drops with LVDD type when compared to control, due to reduced flow during abnormal diastole. In R-type LVDD, the greater degree of systolic dysfunction due to increased septal stiffness has an additional effect on the nature of pulmonary venous flow (Figure 5B4). Percent variation from control is shown in parentheses.

### Summary of Right Heart Effects

Diastolic dysfunction of the LV has notable effects on the right heart. As described in detail above, the E/A wave ratio for tricuspid flow with LVDD is consistently below 1, unlike mitral flow, and increasing in severity in the order IR→R→PN (Figure 4, Figure 5, and Figure 6). Similarly, the D/S ratio of atrial inflow consistently drops in the same order of severity in the RA, unlike the LA with positive change in R-type LVDD (Table 4). In addition, while the LV is marked by normal EF particularly with increased systolic contractility, these studies indicate that with normal contractility from a control value of 0.62 (Table 2), impaired relaxation decreases RVEF to 0.49, restrictive filling decreases it to 0.44, and the combined abnormalities decrease it further to 0.37.

Septal dysfunction with LVDD has effects on the right heart. The septal role in RV ejection is lost with diminished septal priming, delaying opening of the pulmonic valve. Reduced contractility also changes the morphology of ventricular pressure waveforms, with loss of normal trends in systolic P_RV _and P_LV_.

### LVDD with Normal and Abnormal Septal Stiffness

**Figure 10:**
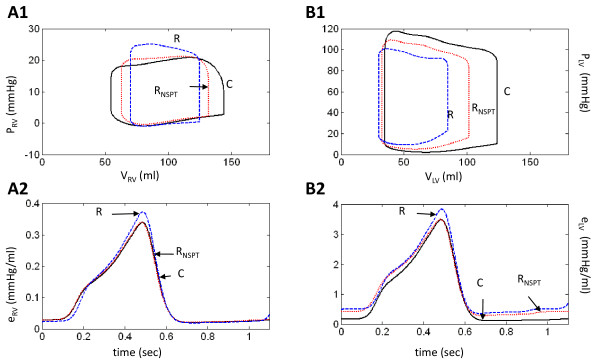
**Model Ventricular Function Curves - Septal Stiffness Comparison**. Simulated ventricular function curves of normal physiology (control, C), increased LV wall *and *septal stiffness (R), and increased LV wall but *normal *septal stiffness (R_NSPT_). All simulations performed with normal systolic contractility.

In the P-V loops of Figure 10A1 and 10B1, the curve labeled R simulates R-type LVDD with elevated levels of stiffness for both the free wall and septum (as in Figure 3). The curve labeled R_NSPT _represents a second simulation where the septal stiffness is set to normal control levels, all other conditions being the same. Focusing on the LV ejection phase of the P-V loops in Figure 10B1, the simulated progression of septal disease R_NSPT _→ R causes the septum to support free wall pumping to a lesser degree, diminishing the "ramping up" of LV pressure during the ejection phase and reducing stroke volume. Changes in septal stiffness also have a pronounced effect on the P-V loops of the RV (Figure 10A1). The ejection phase is downward in the P-V loop in control. With increased LV wall and then septal stiffness, this slope changes to upward, indicative of the increased afterload imposed on the ejecting RV.

Figure 10B2 shows the LV elastance curves for the two cases of R_NSPT_-type and R-type LVDD. Both restrictive cases exhibit a diastolic offset in elastance relative to control. Peak LV elastance in R_NSPT_-type LVDD is at control levels, whereas it is elevated in R-type LVDD. In the case of the RV, there is no diastolic offset in elastance, the R_NSPT _and control elastance curves are nearly identical, and the R elastance curve is elevated by a baroreflex-mediated increase in myocardial contractility. The LV is affected in the same way.

### LVDD with Increased Systolic Contractility

Recent literature [2,3,17,18] suggests that increases in systolic contractility can reduce the end-systolic volume of ventricles affected by diastolic dysfunction and so compensate for the decreased stroke volume caused by the smaller end-diastolic volume. Data from LVDD patients [1] indicates that chronic tissue changes that occur in response to abnormalities such as increased pressure and volume loads can affect myocardial force generation as well as passive transmission of force through the ventricular wall. In this case, we assume that changes in the EDPVR in the free wall or septal component of the model are accompanied by an increase in the corresponding ESPVR characteristic. The usual inotropic factors (α(F_con_) in Eqn. 2) are also at play in the case of baroreceptor-mediated increases in ventricular contractility that occurs in response to changes in MSAP.

**Figure 11:**
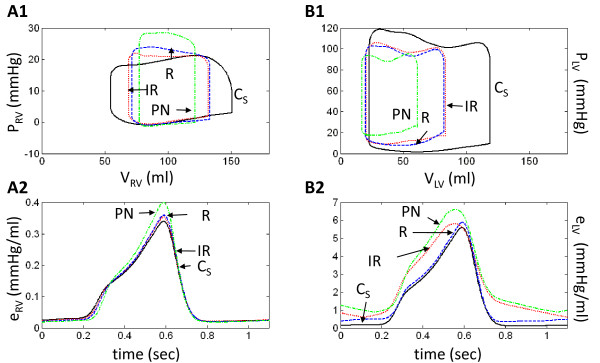
**Model Ventricular Function Curves - Increased LV Systolic Contractility**. Model-generated ventricular function curves of LVDD *with *increased LV systolic contractility. Abbreviations are as in Figure 3. C_S _represents a new elastance control curve where the ESPVR contractility has been augmented, but no other changes have been made.

Considering only simulations of IR-type LVDD, adding increased ESPVR contractility decreases both LV end-systolic and end-diastolic volumes. The new loop produced has the same shape, but is shifted leftward toward lower volumes (compare the IR simulations of Figure 3B and Figure 11B1). The shift produces relatively little change in stroke volume, cardiac output, arterial pressure, or heart rate (Table 2 and Table 3). The LV elastance curve however, has a pronounced diastolic component due to impaired relaxation and its peak is elevated with the induced increase in ESPVR contractility (compare Figure 8B and Figure 11B2). LV ejection fraction increases from 0.68 to 0.76. The control waveform in Figure 11B2 (labeled C_S_) incorporates the increase in ESPVR contractility, but all other parameters are unchanged. Its peak magnitude is therefore considerably larger than that of the normal control waveform. The RV ejection fraction remains approximately the same with the increase in LV systolic contractility and F_con_, although slightly elevated relative to control (0.40-0.45), remains relatively constant (0.46). The RV elastance curve is relatively unaffected by increasing ESPVR contractility (compare IR-type LVDD curves in Figure 8A and Figure 11A2) and is quite similar to normal control (C).

One obtains slightly different results by adding increased LV ESPVR contractility to simulations of R-type LVDD (compare the R P-V loops in Figure 3B and Figure 11B1; Table 2 and Table 3). Here, mean systemic arterial pressure (MSAP) increases from 89.0 to 90.6 mmHg, cardiac output from 3.5 to 3.9 L/min and LVEF from 0.65 to 0.76. The LV elastance curve in the R-type LVDD simulation has a diastolic offset (Figure 11B2) that is relatively constant and quite unlike the time-varying diastolic component of the IR LV elastance curve. F_con _is slightly decreased (0.49 to 0.47) but elevated relative to control C_S _of 0.38. The RV elastance curve in R-type LVDD shows that this increase in LV systolic contractility has virtually no effect on the RV elastance function (compare Figure 8A and Figure 11A2; Table 2).

Increasing the LV ESPVR contractility in PN-type LVDD does not change LV function significantly, other than by more modestly increasing LVEF from 0.63 to 0.72, a number consistent with Kawaguchi's report (70.3 ± 14.8%) (1). LV stroke volume in PN with systolic augmentation is essentially the same as in the original PN-type LVDD case (43.5 compared to 44.1 ml). Table 2 indicates that F_con _levels for the PN case do not change as well. The LV elastance curve in PN has a time-varying diastolic component and an elevated systolic peak (Figure 11B2). Since baroreflex-mediated F_con _levels do not change due to systolic augmentation, the elevated peak of the LV elastance curve (Figure 11B2) is explained simply as the C_S _control systolic elastance component being moved upward by the elevated time-varying diastolic component (i.e., a movement upward toward increased LV elastance (time-varying stiffness)). A comparison of Figure 8A and Figure 11A2 for PN-type LVDD shows that the time course of the RV elastance curves is essentially the same with and without LV systolic augmentation. We note however, that increasing the systolic contractility of an LV afflicted with any form of LVDD does not normalize pulmonary pressures or volumes; therefore pulmonary congestion persists (Table 3).

### Effects on Left Atrial Performance

**Figure 12:**
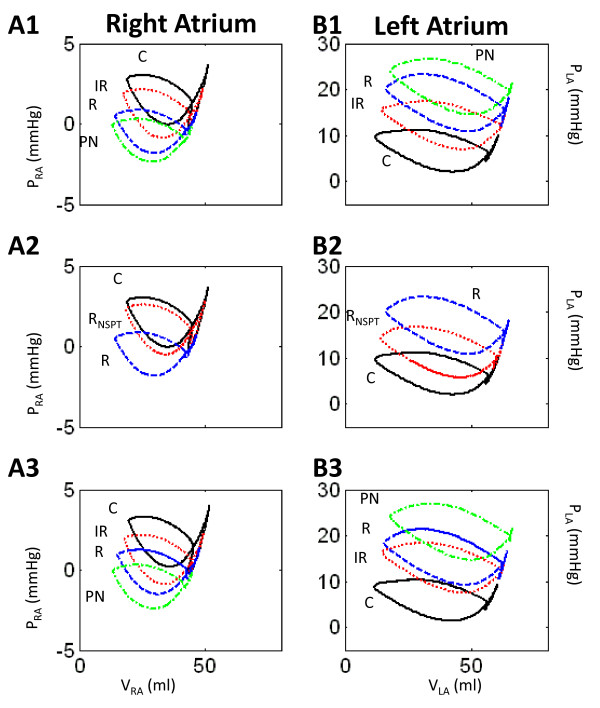
**Comparison of Atrial P-V Loops**. Comparison of normal atrial P-V loops (C, solid line) with those occurring in various types of LVDD. In Panels 1 and 2, LV systolic contractility is normal, whereas in Panel 3, systolic contractility is increased. Panel 2 compares a normal (R_NSPT_) with a stiffened (R) septum in R-type LVDD. Other abbreviations are as in Figure 3.

Figure 12 shows the effect of the different types of LVDD on the instantaneous pressure-volume loops of the right and left atria. Figure 12A1 and B1 show the effects of the three types of LVDD on P-V characteristics of the right and left atria, respectively for the case where the LV has normal ESPVR contractility. In the LA, there is a shift upward and to the right toward higher values of pressure and volume (size) in the simulation sequence C → IR → R → PN (Figure 12B1), whereas RA pressures and volume decrease in the same sequence (Figure 12A1). An increase in the size of the LA relative to control is a common finding in various types of LVDD. In a study on 276 patients, Park et al. [19] have shown that the severity of LVDD correlates well with left atrial dimensions. As the degree of LVDD became more severe, left atrial size and volume increased.

Figure 12A2 and 12B2 examine only the restrictive LVDD case of either normal septal stiffness (R_NSPT_-type) or increased stiffness associated with R-type LVDD. In the LA, the P-V loop is displaced upward and to the right in the simulation sequence C → R_NSPT _→ R in nearly equal increments in pressure and volume. However in the RA, the loops are displaced downward and to the left, but not in equal increments. With normal septal stiffness, the RA P-V loop is very similar to the control loop. However, with the increased septal stiffness inherent in R-type LVDD, the P-V loop is strongly depressed. The difference here is in septal contractile capability, which is strongly curtailed in R-type LVDD (Figure 7A3). Thus, septal integrity is very important to RA performance as it is to RV pumping. With increased LV systolic contractility, there is very little difference between the RA and LA P-V loops shown in Figure 12A3 and 12B3 and Figure 12A1 and B1, respectively.

### Effect of Respiratory Variation

**Figure 13:**
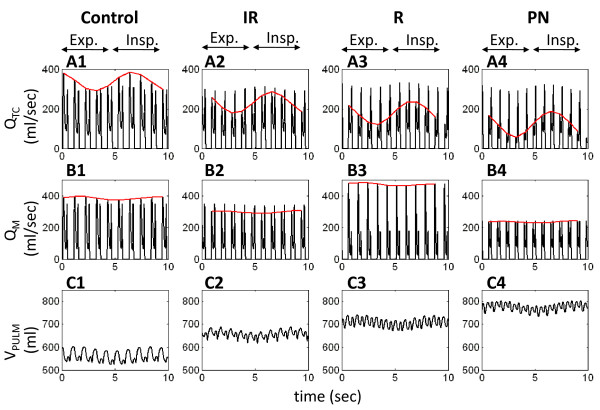
**Influence of Respiratory Variation**. Transvalvular flow variation (Q_TC _shown in Panels A1-A4 and Q_M _shown in Panels B1-B4) during a cycle of respiration (expiration (Exp.) and inspiration (Insp.) are marked). The red lines trace the respiratory variation. Pulmonary vasculature volume is shown in Panels C1-C4. Pulmonary blood volume increases with LVDD, with IR-type LVDD having the lowest increase and PN-type LVDD having the highest increase.

Pleural pressure affects cardiac flows, commonly observed as variation in transvalvular flows coincident with respiration. In a healthy individual, inspiration causes an increase in systemic inflow, increasing Q_TC _in comparison to Q_TC _during expiration. As a result, this variation in systemic inflow is carried across through the pulmonary circulation to the left heart inflow, whereby 2-3 heartbeats later, (roughly coincident with expiration) Q_M _is at a maximum, and during inspiration Q_M _is at its minimum [20]. The model respiratory waveform used in this study is roughly sinusoidal, varying from -2 to -6 mmHg over a 7-second period, and has been used in previous studies [7,8,11]. Our simulations show that the percent respiratory variation (percent deviation from maximum flow) in control Q_TC _is 24.2% and 5.5% in Q_M _(Table 5 and Figure 13A1 and 13B1). In LVDD, respiratory variation in Q_TC _becomes much more pronounced, with values of 36.9%, 48.1% and 70.1% for the IR, R and PN cases, respectively (Table 5 and Figure 13A2-A4). Respiratory influence on mitral flow Q_M _is weak, but can be seen in the control case (Figure 13B1). In LVDD, there is a progressive reduction in percent respiratory variation in Q_M _in the direction IR → R → PN LVDD (Table 5 and Figure 13B2-B4). Concurrently, pulmonary blood volume increases in the same direction of IR → R → PN LVDD (Figure 13C2-C4), acting as a buffer against left heart respiratory variation. This increase in pulmonary blood volume is accompanied by increased afterload on the RV and hence RV pressure increases (Figure 3A). The buffering effect of the pulmonary blood volume seemingly decouples the respiratory variation so that it mainly affects the right heart as RV systolic pressures increase and diastolic pressures decrease, becoming even more influenced by P_PL _and less influenced by the septum. Moreover, the mean position of the septum is displaced rightward in IR-type, and leftward in R-type and PN-type LVDD (Figure 7A3), with attendant loss of pumping efficiency in all LVDD cases relative to control.

**Table 5:** Percent Respiratory Variation for Various Flows and Volumes

	Control	IR	R	PN
**Q**_**TC**_	24.2%	36.9%	48.1%	70.1%

**Q**_**M**_	5.5%	5.3%	4.5%	3.7%

**V**_**RV**_	14.5%	15.2%	17.9%	18.8%

**V**_**PA,p**_	6.1%	7.0%	6.1%	6.4%

**V**_**PA**_	6.6%	6.5%	5.7%	5.9%

**V**_**PA,d**_	6.1%	7.0%	6.1%	6.4%

**V**_**PC**_	4.8%	4.0%	4.1%	3.6%

**V**_**PV**_	1.7%	2.6%	2.5%	2.8%

**V**_**LV**_	3.4%	3.1%	1.5%	2.5%

Percent respiratory variation between inspiration and expiration for various flows and volumes in the control and LVDD cases.

## Discussion

Several factors can interact to cause LV diastolic dysfunction, increasing the difficulty of identifying mechanism(s) underlying any one case. It would help if one could isolate and independently change each putative cause of LV diastolic dysfunction, and then study the subsequent effects of each. This study and others we have published show that by changing just a few parameters of our H-CRS model, one can closely approximate the myriad effects of LVDD associated with congestive heart failure. The complexity of the model allows for an accurate and comprehensive view of the problem. Here, LV active relaxation and passive wall stiffness are each represented by a single parameter, and changing one or both closely simulates many of the abnormalities seen in patients with LVDD. Simulating LVDD with this model is straightforward and appears to be an excellent means of addressing the controversies surrounding HFNEF causation.

Because pulmonary pressures increase and the A-V O_2 _difference widens as stroke volume, cardiac output, and mean arterial pressure decrease, the model confirms that LVDD is a form of heart failure, since cardiac function cannot match the metabolic requirements of the body, or can do so only at elevated LV filling pressures. The increase in pulmonary blood volume and efferent sympathetic nervous system activity are also typical of heart failure. The model reproduces these pathologic features without decreasing LVEF to a number that clinicians would regard as significantly depressed, and so demonstrates that LVDD alone can cause most (but perhaps not all) the major signs of HFNEF.

In the three types of LVDD we modeled, pulmonary pressures and volumes were all elevated (Table 3). They were reduced but not entirely normalized by increasing LV contractility, since persistent LA pressure elevation (Figure 12B1) would maintain high pulmonary venous, capillary, arteriolar, and pulmonary arterial pressures. Dyspnea would probably result if R-type LVDD brought the LVEDP to 23.0 mmHg, and even more likely with an LVEDP of 25.0 mmHg in PN-type LVDD (Table 2). This would be especially true if these increases were new, and not offset by the previous, gradual increase in lymphatic removal of lung edema that occurs with longstanding LVDD. Currently our H-CRS model does not contain a lung lymphatic drainage model, and cannot be used to predict how gas exchange would be affected in longstanding LVDD.

Removing neural feedback for cardiac contractility and heart rate shows that augmentation of these aspects in the right heart with LVDD is purely neural. In the left heart neural augmentation plays a role in increasing peak elastance, improving early systole in R-type LVDD, and increasing heart rate, leaving altered elastance curves mainly a result of LVDD.

Recent literature [1,2] states that if LV systolic contractility is increased, the reduced end-systolic volume that is obtained can partly compensate for the reduced end-diastolic volume produced by LVDD, although cardiac output remains decreased. It indicates that chronic tissue changes, and not just greater sympathetic traffic, increase ESPVR stiffness. This is consistent with findings that concentric LV hypertrophy increases both passive stiffness and systolic contractility [1,17]. Although LVEF is sustained (or even supernormal), diastolic LV pressures, pulmonary pressures, pulmonary blood volumes, and heart rates remain elevated. The circulation is adequate under these conditions, but is maintained at the expense of elevated filling pressures and pulmonary congestion, which often provokes dyspnea and reduces exercise tolerance.

LVDD is often detected by recording abnormalities in the diastolic flow across the AV valves during early rapid filling and atrial systole, and an abnormal E/A ratio can also suggest LVDD. But the diagnosis may be missed if restrictive filling and impaired relaxation combine to pseudo-normalize the E/A ratio, which occurs if left atrial and pulmonary pressures are severely elevated, and diminished blood return lowers right atrial and central venous pressure. Consequently, it may be difficult to detect or determine the cause of LVDD using only LV measurements. Our model suggests that in this situation, right heart function might provide diagnostic clues. For example, the tricuspid flow pattern remains abnormal when mitral flow is pseudo-normalized, and the right ventricular ejection fraction is always abnormal even though LV systolic contractility is increased. Perhaps the term HFNEF should only apply to the LV, since the right ventricular ejection fraction is never normal in the presence of LVDD.

Septal role in hemodynamics is limited with LVDD. The loss of septal priming motion diminishes septal aid to RV ejection delaying opening of the pulmonic valve and altering the endpoint of RV outflow as well. The stiffened septum in R-type and PN-type has a slower leftward stroke contributing less to LV ejection. Abnormal septal performance produces changes to the mechanical synchrony of ventricles during systole.

The model predicts that either active relaxation or passive stiffness, or both, will increase A-V differences across the lung, systemic, and cerebral tissues (Table 2), and that these differences correlate linearly with decreases in mean arterial pressure and cardiac output. Our simulations show that impairing either active relaxation or passive stiffness creates nearly identical decreases in cardiac output, and the changes in A-V O_2 _and CO_2 _concentrations are likewise similar. Much larger A-V differences result when both active relaxation and passive stiffness are abnormal. Increasing systolic contractility does not reverse these changes. Cerebral autoregulation stabilizes brain perfusion despite widely varying cardiac outputs, and the resulting changes in O_2 _extraction and CO_2 _deposition are more narrowly confined. The model predicts that when extra-cerebral O_2 _and CO_2 _differences widen by as much as 16.9 and 6 mmHg, respectively, they will increase in the brain by less than 5.1 and 3.9 mmHg, respectively.

In summary, the following occur in any form of isolated LVDD (in the absence of a compensatory increase in total body fluid volume):

1. elevated LVEDP

2. reduced stroke volume and cardiac output; increased A-V O_2 _and CO_2 _differences

3. reduced tricuspid flow E/A ratio

4. prolonged tricuspid flow deceleration time

5. wider pulmonary venous flow (PVF) S1 S2 separation with reduced S2

6. decreased central venous flow (CVF) D/S ratio

7. decreased central venous pressure

8. increased pulmonary venous pressure

9. decreased RVEF

In addition, restrictive filling can:

1. increase mitral flow E/A ratio

2. shorten mitral flow deceleration time

3. increase pulmonary venous flow D/S ratio,

while impaired LV free-wall relaxation can:

1. decrease mitral flow E/A ratio

2. prolong mitral flow deceleration time

3. decrease pulmonary venous flow D/S ratio

Finally, combining impaired relaxation and restrictive filling shows:

1. a normal mitral flow E/A ratio

2. a normal deceleration time

The model shows that the opposing flow waveforms of combined impaired relaxation and restrictive filling will "compete" to shape the final mitral inflow pattern. Just how "normal" a pseudo-normalized pattern becomes will depend on the dominant mechanism. But again, RV function should be a less ambiguous indicator of LVDD, since both the pulmonary vein and tricuspid flow patterns remain abnormal despite a pseudo-normal mitral flow pattern. A suggestive tricuspid E/A ratio combined with evidence of elevated LVEDP and pulmonary congestion could be more diagnostic of LVDD than the more traditional mitral E/A measurement.

In short, this modeling study confirms several experimental findings. Firstly, we demonstrate that LVDD causes heart failure, with commonly recognized signs of decreased cardiac output, stroke volume, and mean arterial pressure, A-V O_2 _difference widening, and pulmonary congestion. Secondly, we show that normal ejection fraction occurs with increased LV systolic contractility (a result of experimentally observed chronic tissue changes), producing the well-known HFNEF phenomenon. Importantly, our modeling study points out key features of LVDD not previously recognized. These include: (a) the consistent right heart signs of LVDD, e.g., decreased E/A wave ratios regardless of LVDD type (Figure 4, Figure 5, and Figure 6); (b) the pronounced effect of changes in septal motion on RV mechanics (Figure 7-Figure 8); and (c) the effects of neural augmentation of RV contractility on RV mechanics (Figure 9).

### Limitations

All models have limitations and some of the more important limitations associated with the current study are listed below.

(1)LVDD alone may not produce every defining sign of HFNEF. This study has focused on abnormal diastolic properties of the left ventricle, and not evaluated how extra-cardiac pathology such as reduced arterial compliance [21,22] might contribute to the syndrome. The diastolic changes introduced to model the various types of LVDD were made to mimic acute LVDD in the human patient on a short time scale. Longer term adjustments by the body are neglected including chronic changes in blood volume and venoconstriction. The model leaves one suspecting that such factors are operative, however, at least in some cases.

(2)In our LVDD simulations, we have induced model parameter changes that impair active relaxation or increase wall stiffness of the LV (IR and R cases). Individually, these modeled changes in LV mechanics were shown to have a nearly equal effect on the cardiovascular system, and with intermediate severity compared to both effects acting together as in PN-type LVDD. With these simple assumptions, we were able to characterize the three main types of LVDD with changes in hemodynamic severity in the direction C → IR → R → PN. Of course, with other weightings of IR or R disease, this progression of LVDD severity could change. Nevertheless, these simulations with simple assumptions have emulated the classical clinical classifications of LVDD. The important contributions of this work lie however, in the mechanistic explanations of these different disease entities particularly in elucidating the role of septal mechanics in each case. This work would be much enhanced by the availability of patient hemodynamic data sets that would include bi-ventricular high fidelity pressure recordings and transvalvular Doppler flow velocity recordings from the tricuspid and mitral valves. Ultimately, modeling work of this type should be directed toward the hemodynamic characterization of the individual patient. The question arises that if a patient whose LVDD is the result only of increased stiffness (R), as may be the case at low heart rates [2], would that alone be sufficient to produce all the resulting signs and symptoms of heart failure? It is our hope that future versions of our H-CRS model could not only characterize the patient's ventricular mechanics, but could also incorporate the additional extra-cardiac factors that might help answer this question.

(3) In planning for more comprehensive studies of congestive heart failure, the model will require an update on the lung lymphatic drainage model currently used. In addition, patients with congestive heart failure often exhibit Cheyne-Stokes respiration (characterized by a periodic waxing and waning of the respiratory tidal volume). This is a most interesting problem, actually a separate study in and of itself, that would have impact on gas transport, autonomic control of the cardiovascular system and introduction of hemodynamic variations that could have consequences on ventricular mechanics. Although this is a very appropriate topic for analysis by our H-CRS model, it is beyond the scope of the current manuscript and therefore we leave it to future work.

## Conclusions

Adjustment of a few parameters that determine the LV mechanics of our human cardiovascular-respiratory system model simulates many of the hemodynamic and respiratory features of LV diastolic dysfunction. This larger model is superior to one limited to the LV alone because it reproduces the global response to any change in LV mechanics and provides a biophysical explanation of many clinical findings. Our simulations show that both restrictive filling and impaired relaxation cause LVDD. In combination, these conditions pseudo-normalize the mitral E/A ratio even though the LV and especially the RV ejection fractions are reduced. An increase in contractility can compensate for the reduction in the LV ejection fraction, but would not reduce pulmonary pressures or blood volume, and so pulmonary congestion would persist. The important role of the septum in RV systolic ejection is reduced. And although HFNEF is a possible indicator of LVDD, a correct diagnosis may be missed if only LVEF and other LV function indices are considered. Rather, the model results suggest that changes in RV function may demonstrate unique features that may significantly aid the diagnosis.

## List of Abbreviations

### Cardiovascular Model

RA: right atrium; LA: left atrium; RV: right ventricle; LV: left ventricle; RVF: RV free wall; LVF: LV free wall; A-V: arterio-venous; ESPVR: end-systolic pressure-volume relationship; EDPVR: end-diastolic pressure-volume relationship; P_TMAO,p_: transmural aortic pressure (proximal); P_TMAO,d_: transmural aortic pressure (distal); P_SA,d_: systemic arteriole pressure (distal); P_SC_: systemic capillary pressure; P_SVL_: systemic venule pressure; P_SV_: systemic venous pressure (distal); P_VC_: systemic venous pressure (proximal) or vena caval pressure; P_TMPA,p_: transmural pulmonary arterial pressure (proximal); P_TMPA,d_: transmural pulmonary arterial pressure (distal); P_PA_: lumped pulmonary arteriolar pressure; P_PC_: pulmonary capillary pressure; P_PV_: pulmonary venous pressure; P_LV_: LV pressure; P_AO,p_: aortic pressure (proximal); P_AO,d_: aortic pressure (distal); P_LA_: LA pressure; P_RV_: RV pressure; P_PA,p_: pulmonary arterial pressure (proximal); P_PA,d_: pulmonary arterial pressure (distal); P_RA_: RA pressure; P_SPT_: trans-septal pressure; P_PERI_: pericardial pressure; P_LVF_: LVF pressure; P_RVF_: RVF pressure; P_x,ES_: pressure of x at end-systole (where x: LV,RV,LA,RA); P_x,ED_: pressure of x at end-diastole (where x: LV,RV,LA,RA); P_x,0_: nominal diastolic pressures for x (where x: LVF,RVF,SPT,LA,RA); V_LV_: LV volume; V_AO,p_: aortic volume (proximal); V_AO,d_: aortic volume (distal); V_SA_: lumped systemic arteriolar volume (proximal); V_SA,d_: lumped systemic arteriolar volume (distal); V_SC_: systemic capillary volume; V_SVL_: lumped systemic venules volume; V_SV_: systemic venous volume (distal); V_VC_: systemic venous volume (proximal) or vena caval volume; V_LA_: LA volume; V_RV_: RV volume; V_PA,p_: pulmonary arterial volume (proximal); V_PA,d_: pulmonary arterial volume (distal); V_PA_: pulmonary arteriolar volume; V_PC_: pulmonary capillary volume; V_PV_: pulmonary venous volume; V_RA_: RA volume; V_SPT_: septal volume; V_LVF_: LVF volume; V_RVF_: RVF volume; V_x,d_: zero-pressure volume for the systolic pressure relationship (where x: LVF,RVF,SPT,LA,RA); V_x,0_: zero-pressure volume for the diastolic pressure relationship (where x: LVF,RVF,SPT,LA,RA); Q_AO,d_: aortic flow (distal); Q_SA_: systemic arterial flow (proximal); Q_PA,d_: pulmonary arterial flow (distal); Q_PA_: pulmonary arteriolar flow; Q_LA_: left atrial inflow; Q_M_: mitral flow; Q_AO,p_: aortic flow (proximal); Q_SA,d_: systemic arteriolar flow; Q_SC_: systemic capillary flow; Q_SVL_: lumped systemic venules flow; Q_SV_: systemic venous flow (distal); Q_VC_: systemic venous flow (proximal) or vena caval flow; Q_RA_: RA inflow; Q_TC_: tricuspid flow; Q_PA,p_: pulmonary arterial flow (proximal); Q_PS_: pulmonary A-V shunt flow; Q_PC_: pulmonary capillary flow; Q_PV_: pulmonary venous flow; Q_COR_: coronary flow; E_LVF_: LVF elastance; E_LA_: LA elastance; E_RVF_: RVF elastance; E_RA_: RA elastance; e_x(t)_: time-dependent activation function of x (where x: LVF,RVF,RA,LA); E_x,ES_: slope of linear ESPVR of x (where x: LVF,RVF,SPT,LA,RA); α(F_con_): dimensionless neural control factor; λ_x_: stiffness parameter associated with the passive diastolic pressure relationship (where x = LV,RV,SPT,LA,RA); R_M_: mitral valve resistance; R_AO,p_: aortic valve resistance; R_COR_: coronary arterial resistance; R_NA_: neck arterial resistance; R_SA,d_: systemic arterial resistance (distal); R_SC_: systemic capillary resistance; R_SVL_: systemic venules resistance; R_SV_: systemic venous resistance; R_VC_: vena caval resistance; R_RA_: RA influx resistance; R_TC_: tricuspid valve resistance; R_JV_: jugular venous resistance; R_PA,p_: pulmonary arterial resistance (proximal); R_TPA_: transmural pulmonary arterial resistance; R_PC_: pulmonary capillary resistance; R_PS_: pulmonary arterial to venous shunt resistance; R_PV_: pulmonary venous resistance; R_LA_: LA influx resistance; C_AO,p_: aortic arterial compliance (proximal); C_AO,d_: aortic arterial compliance (distal); C_SA,d_: systemic arterial compliance (distal); C_SC_: systemic capillary compliance; C_SVL_: systemic venules compliance; C_PA,p_: pulmonary arterial compliance (proximal); C_PA,d_: pulmonary arterial compliance (distal); C_PA_: pulmonary arterial compliance; C_PC_: pulmonary capillary compliance; C_PV_: pulmonary venous compliance; L_AO,p_: aortic arterial inertance (proximal); L_AO,d_: aortic arterial inertance (distal); L_AO,d_: aortic arterial inertance (distal); L_PA_: pulmonary arterial inertance;

### Pulmonary and Gas Exchange Model

P_j _^i^: pressure in region j of species i, where i: O_2_, CO_2_, or both (*), and j = alveoli (A), collapsible airways (C), rigid dead space region of airways (D), interstitial space (IS), intracellular space (IC), or atmosphere (ATM); P_EL_: alveolar transmural pressure; P_TM_: collapsible airway transmural pressure; P_S_: standard pressure; V_A_: pulmonary alveolar volume; V_C_: pulmonary collapsible airway volume; V_CW_: total gas volume in lungs; V_VE_: lung viscoelastic volume; V_D_: anatomic dead space volume; V_C,max_: maximum collapsible airway volume; V*: alveolar volume at end inspiration; Q_ED_: air flow in upper airways; Q_CA_: airflow between collapsible airway and alveolar space; Q_DC_: airflow between dead space and collapsible airways; R_C_: collapsible airways resistance; R_S_: small airways resistance; R_Sc_: R_S _at V*; R_Sm_: magnitude of (R_S_-R_Sc_) at minimal alveolar volume; R_Sa_: parameter characterizing curvature of R_S_; C_j _^i ^: concentration in region j of species i, where i = O_2 _or CO_2_, and j = IS or IC; P_PL_: pleural pressure; φ_tot,j_^i ^: total rate of transfer of species i in region j, where i = O_2_, CO_2_,or both (*), and j = lung (L), tissue (T), or brain (B); T_body_: body temperature; T_S_: standard temperature; α_i_: tissue disassociation constant of species i, where i = O_2_, CO_2_; D_j_^i^: diffusing capacity of species i in region j, where i = O_2_, CO_2 _and j = membrane (MEM), brain (B), or CSF; M_j_^i^: metabolic rate of species i consumption in region j, where i = O_2_, CO_2_, and j = tissue (T) or brain (B); K_myo_: myoglobin capacity of O_2_; K_1_: linear resistance of upper airways; K_2_: flow-dependent resistance of upper airways; K_3_ = magnitude of R_C _at V_C_: V_C,max_; N_seg_: number of capillary segments;

### Tissue Water Exchange and Lymphatics Model

P_LYM_: systemic lymphatic pressure; P_IC_: intracellular fluid pressure; V_IS_: interstitial fluid volume; V_IC_: intracellular fluid volume; V_LYM_: systemic lymphatic volume; P_IS_: interstitial fluid pressure; Q_LYM,d_: systemic lymphatic flow (distal); Q_LYM,p_: systemic lymphatic flow (proximal); Q_F,tot_: total water flux across capillary; Q_IC_: intracellular fluid flow; R_IC_: intracellular flow resistance; K_F_: filtration coefficient;

### Neural Model

F_b_: baroreceptor frequency; F_s_: pulmonary stretch receptor frequency; F_c_: chemoreceptor frequency; F_cc_: central chemoreceptor frequency; F_HRs_: normalized frequency of sympathetic control of HR; F_HRv_: normalized frequency of vagal control of HR; F_con_: normalized sympathetic efferent discharge frequency controlling contractility; F_vaso_: normalized sympathetic efferent discharge frequency controlling vasomotor tone; K_f_: baroreceptor neural parameter; θ_s_: for low pass filtering of pulmonary stretch receptor frequency; E_cl_: for low pass filtering of peripheral chemoreceptor signal; E_cc_: for low pass filtering of central chemoreceptor signal; E_HRv_: for low pass filtering of vagal control of HR signal; E_HRs_: for low pass filtering of sympathetic control of HR signal; E_con_: for low pass filtering of contractility signal; E_vaso_: for low pass filtering of vasomotor tone signal; K_chm_: chemoreceptor variable;

### Cerebral Circulation and Gas Exchange Model

CSF: cerebral spinal fluid; P_NA_: neck arterial pressure; P_CA_: cerebral arterial pressure; P_CC_: cerebral capillary pressure; P_ICR_: intracranial pressure; P_j_^i^: pressure in region j of species i, where i = O_2_, CO_2_, or both (*), and j = CSF, BIS, or BIC; P_NV_: neck venous pressure; P_CV_: cerebral venous pressure; V_NA_: neck arterial volume; V_CA_: cerebral arterial volume; V_CC_: cerebral capillary volume; V_CV_: cerebral venous volume; V_NV_: neck venous volume; V_ICR_: intracranial volume; V_BS_: brain interstitial tissue volume; V_BC_: brain intracellular tissue volume; C_j_^i^: concentration in region j of species i, where i = O_2_, CO_2_, or both (*), and j = CSF, brain interstitial (BIS), or brain intracellular (BIC); Q_NA_: neck arterial flow; Q_CA_: cerebral arterial flow; Q_CC_: cerebral capillary flow; Q_CV_: cerebral venous flow; Q_JV_: jugular venous flow; Q_F_: CSF formation rate; Q_0_: CSF absorption rate; R_NA_: neck arterial resistance; R_CA_: cerebral arterial resistance; R_CC_: cerebral capillary resistance; R_CV_: cerebral venous resistance; R_JV_: jugular venous resistance; R_F_: CSF formation resistance; R_0_: CSF absorption resistance; C_CA_: cerebral arterial compliance; x_aut_: cerebral autoregulation variable;

## Competing interests

The authors declare that they have no competing interests.

## Authors' contributions

CL carried out the primary modeling studies and drafted the manuscript. DR contributed to the analysis of model data and manuscript writing. DLW and TSM made substantial intellectual contributions to the study and in drafting of the manuscript. JWC made key contributions to the conception and design, analysis and interpretation of data, and drafting of the manuscript. All authors read and approved the final manuscript.

## Appendix A

Model equations are provided below. See Appendix B (Table 6, Table 7, Table 8, and Table 9) for parameter values.

### Cardiovascular Model

The ordinary differential equations to compute pressures, volumes, and flows in the circulatory loop are as follows. Model description of ventricular pressures is given in the Methods section, and repeated below for convenience.

The instantaneous pressure (mmHg) within either V_LVF _or V_RVF _is the weighted sum of pressure during diastole and systole [5]:

where

and

Since both P_LVF _and P_RVF _are transmural (differential) pressures with reference to P_PERI_, the absolute chamber pressures P_LV _and P_RV _(relative to atmosphere) are equivalent to the respective free wall transmural pressure plus P_PERI_. LA and RA are described similarly.

The trans-septal pressure difference (mmHg) is:

V_SPT _is calculated from the difference in the two free wall pressures, and is the weighted sum of diastolic and systolic contributions.

If P_SPT _≥ 0,

If P_SPT _< 0,

where E_SPT,ES_ = 40 mmHg/ml, V_SPT,d_ = 0 ml, λ_SPT_ = 0.05 ml^-1^, P_SPT,0_ = 1.11 mmHg, V_SPT,0_ = 0 ml

Septal volume is then the weighted sum of septal volume in systole and diastole:

Given the model storage element volumes (V_LVF_, V_RVF _and V_SPT_), the corresponding transmural pressures for the free walls and septum can be calculated. Total V_LV _and V_RV _are defined as:

In these equations e_x_(t) is the dimensionless weight or "activation function," denoting myocardial activation as between 0 and 1, where x = LVF, RVF, or SPT. Ventricular mechanics is described by separate mechanical and temporal behavior - mechanical behavior by static free wall pressure-volume characteristics and temporal behavior by e_x_(t) functions.

The circulatory loop is computed as follows, beginning with the LV:

where

where P_body _is equal to P_IS _(see below)

(see below for Q_F,tot_)

(see below for P_PL_)

(see below for Q_LYM,p_)

(see below for P_A_*)

### Lung and Airways Mechanics Model

The airways model consists of the upper rigid dead space region, collapsible mid-airways region, and the lower small airways region. The rigid upper airway is characterized by a flow-dependent resistor (Rohrer resistor), where airflow is given as:

Partial pressures in upper airways are given by:

A nonlinear P-V relationship characterizes the collapsible mid-airways with transmural pressure P_TM _given by:

where

The collapsible mid-airways volume is as follows, where airflow Q_DC_ = Q_ED_:

Partial pressures in the mid-airways are given by:

The alveolar volume is computed as follows:

The alveolar airflow Q_CA _is:

where

Partial pressures in the alveoli are given by:

Change in chest wall volume is calculated as below:

Lung tissue viscoelasticity is taken into account with volume V_VE_:

Pleural pressure is an approximate sinusoid of period of around 7 seconds (23).

### Systemic Lymphatics and Tissue Water Exchange Model

Systemic lymphatics tap excess fluid from the interstitial space and empty into the systemic venous return:

Intracellular filtration is defined as follows:

Gas concentrations and partial pressures in the intracellular compartment are given by:

Total water flux across the systemic capillaries is determined by the number of capillary segments defined (N_seg_):

Interstitial fluid volume is calculated by:

Gas concentrations and partial pressures in the interstitial compartment are given by:

### Neural Model

Baroreceptor frequency is calculated as follows:

Stretch receptor frequency is calculated as follows:

Peripheral chemoreceptor frequency is calculated as follows:

The signal is low-pass filtered by:

Central chemoreceptor frequency is calculated as follows:

The signal is low-pass filtered by:

Heart rate vagal and sympathetic frequencies are calculated as follows, with low-pass filtering:

Heart contractility frequency and low-pass filtering are calculated as follows:

Frequency for vasomotor tone and low-pass filtering are calculated as follows:

### Cerebral Circulation and Gas Exchange Model

The cerebral circulatory loop is defined as follows:

Gas concentrations and partial pressures in CSF are solved as below:

Gas concentrations and partial pressures in brain interstitial compartment are solved as below:

Gas concentrations and partial pressures in brain intracellular compartment are solved as below:

Cerebral autoregulation is determined as below:

## Appendix B

Model parameters and their values used for the current study.

**Table 6:** Model Resistance Parameters

Mitral Valve (R_M_)	0.007	Pulmonary Arterial Vasoelastic (R_TPA_)	0.035
Aortic valve (R_AO,p_)	0.005	Pulmonary Arteriolar (R_PA_)	0.01

Coronary (R_COR_)	35	Pulmonary Shunt (R_PS_)	4.0

Cerebral (R_CRB_)	16	Pulmonary Capillary (R_PC_)	0.08

Distal Aortic (R_AO,d_)	0.015	Pulmonary Venous (R_PV_)	0.008

Aortic Vasoelastic (R_TAO_)	0.051	LA influx (R_LA_)	0.01

Distal Aortic Vasoelastic (R_TAO,d_)	0.0125	Neck Arterial (R_NA_)	0.8

Systemic Arterial (R_SA_)	0.015	Cerebral Arterial (R_CA_)	1.8

Distal Systemic Arterial (R_SA,d_)	0.35	Cerebral Capillary (R_CC_)	2.8

Systemic Capillary (R_SC_)	0.4	Cerebral Venous (R_CV_)	1.2

Systemic Venules (R_SVL_)	0.2	Jugular Venous (R_JV_)	0.5

Systemic Veins (R_SV_)	0.2	CSF Generation (R_F_)	7500

Vena Cava (R_VC_)	0.026	CSF Absorption (R_O_)	511.6

RA influx (R_RA_)	0.01	Distal Lymphatic (R_LYM,d_)	10

Tricuspid (R_TC_)	0.0028	Proximal Lymphatic (R_LYM,p_)	10

Pulmonary Valve (R_PA,p_)	0.002	Filtration (R_IC_)	1.0

Distal Pulmonary Arterial (R_PA,d_)	0.002		

Model resistance parameters (mmHg.sec/ml)

**Table 7:** Model Compliance Parameters

Aortic (C_AO,p_)	2.4	Distal Pulmonary Arterial (C_PA,d_)	0.64
Distal Aortic (C_AO,d_)	0.43	Pulmonary Arteriolar (C_PA_)	6.0

Systemic Arterial (C_SA_)	0.092	Pulmonary Capillary (C_PC_)	2.1

Distal Systemic Arterial (C_SA,d_)	0.069	Pulmonary Venous (C_PV_)	7.3

Systemic Capillary (C_SC_)	0.8	Neck Arterial (C_NA_)	0.16

Systemic Venular (C_SVL_)	0.6	Cerebral Arterial (C_CA_)	0.12

Systemic Venous (C_SV_)	37.5	Cerebral Capillary (C_CC_)	0.22

Vena Cava (C_VC_)	4.0	Cerebral Venous (C_CV_)	1.0

Pulmonary Arterial (C_PA,p_)	0.69	Systemic Lympatic (C_LYM_)	10

Model compliance parameters (ml/mmHg)

**Table 8:** Model Inertance Parameters

Aortic (L_AO,p_)	0.0055
Distal Aortic (L_AO,d_)	0.0031

Pulmonary Arterial (L_PA_)	0.00008

Model inertance parameters (ml/mmHg/sec^2^)

**Table 9:** Additional Parameters

P_TM,max_	31.379 mmHg	λ_LA_	0.1/ml
P_RV,0_	1 mmHg	λ_RA_	0.1/ml

P_PERI,0_	0.5 mmHg	λ_LV_	0.025/ml

P_LA,0_	2 mmHg	λ_RV_	0.01/ml

P_RA,0_	1 mmHg	λ_SPT_	0.05/ml

P_SPT,0_	1.11 mmHg	λ_PERI_	0.005/ml

P_LV,0_	2 mmHg	R_Sa_	-10.869553 mmHg.sec/ml

P_ATM_^O^_2_	153.12 mmHg	R_VAM_	1360 ml

P_ATM_^CO^_2_	0.30 mmHg	R_R_	0.000735 mmHg.sec/ml

P_PLA_	0.4 mmHg	R_Sm_	2.201863 mmHg.sec/ml

P_LOFF_	-0.5 mmHg	R_Sc_	0.2 mmHg.sec/ml

P_LS_	0.001 mmHg	C_C_	660 ml/mmHg

P_S_	760 mmHg	α(F_con_)	>1

V_LA,d_	7 ml	K_1_	0.003 mmHg.sec/ml

V_RA,d_	3 ml	K_2_	0.3 mmHg. sec^2^/ml^2^

V_LA,0_	40 ml	K_3_	0.00021 mmHg.sec/ml

V_RA,0_	30 ml	K_VC1_	8.0 mmHg

V_SPT,d_	0 ml	K_VC2_	0.05 mmHg

V_LV,d_	1 ml	K_myo_	1.64e-2 ml O_2_/ml blood

V_RV,d_	3 ml	K_f_	1.2

V_SPT,0_	0 ml	K_e_	0.11 ml^-1^

V_LV,0_	1 ml	K_F_	0.033 ml/mmHg.sec

V_RV,0_	3 ml	K_aut_	9.0ml/mmHg

V_PERI,0_	200 ml	C_HCO3_	2.45e-5

V_PERI_	6 ml	τ_aut_	40 sec

V_C,max_	185.46 ml	T_body_	310.16 K

V_CTERM_	40 ml	T_S_	273.16 K

V*	5000 ml	α_O2_	3e-5 mmHg^-1^

V_D_	150 ml	α_CO2_	6.68e-4 mmHg^-1^

V_VC,max_	400 ml	M_T_^O^_2_	3.33 ml/s

V_VC,min_	50 ml	M_T_^CO^_2_	2.83 ml/s

V_VC,0_	130 ml	M_B_^O^_2_	0.77 ml/sec

V_BC_	1150 ml	M_B_^CO^_2_	0.50 ml/sec

V_BS_	210 ml	D_B_^O^_2_	0.38 ml/mmHg.sec

E_LA,ES_	2.5 mmHg/ml	D_B_^CO^_2_	3.33 ml/mmHg.sec

E_RA,ES_	0.34 mmHg/ml	D_MEM_^O^_2_	0.67 ml/mmHg.sec

E_SPT_,_ES_	40 mmHg/ml	D_MEM_^CO^_2_	13.33 ml/mmHg.sec

E_LV,ES_	3.5 mmHg/ml	D_CSF_^O^_2_	0.67 ml/mmHg.sec

E_RV,ES_	0.34 mmHg/ml	D_CSF_^CO^_2_	13.33 ml/mmHg.sec

Additional model parameters

## References

[B1] Kawaguchi M Hay I Fetics B Kass DA Combined ventricular systolic and arterial stiffening in patients with heart failure and preserved ejection fraction: implications for systolic and diastolic reserve limitations Circulation 2003 107 5 714 20 10.1161/01.CIR.0000048123.22359.A0 12578874

[B2] Burkhoff D Maurer MS Packer M Heart failure with a normal ejection fraction: is it really a disorder of diastolic function? Circulation 2003 107 5 656 8 10.1161/01.CIR.0000053947.82595.03 12578861

[B3] Zile MR Baicu CF Gaasch WH Diastolic heart failure - abnormalities in active relaxation and passive stiffness of the left ventricle N Engl J Med 2004 350 19 1953 9 10.1056/NEJMoa032566 15128895

[B4] Lu K Clark JW Jr Ghorbel FH Ware DL Bidani A A human cardiopulmonary system model applied to the analysis of the Valsalva maneuver Am J Physiol Heart Circ Physiol 2001 281 6 H2661 79 11709436 10.1152/ajpheart.2001.281.6.H2661

[B5] Lu K Clark JW Jr Ghorbel FH Ware DL Zwischenberger JB Bidani A Whole-body gas exchange in human predicted by a cardiopulmonary model Cardiovasc Engineering 2003 3 1 19 10.1023/A:1024795417999

[B6] Lu K Clark JW Jr Ghorbel FH Robertson CS Ware DL Zwischenberger JB Bidani A Cerebral autoregulation and gas exchange studied with a human cardiopulmonary model Am J Physiol 2004 286 H584 H601 10.1152/ajpheart.00594.2003 12946929

[B7] Luo C Ware DL Zwischenberger JB Clark JW Using a human cardiopulmonary model to study and predict normal and diseased ventricular mechanics, septal interaction, and atrio-ventricular blood flow patterns J Cardiovasc Engineering 2007 7 17 31 10.1007/s10558-007-9025-9 17334942

[B8] Luo C Ware DL Zwischenberger JB Clark JW A mechanical model of the human heart relating septal function to myocardial work and energy J Cardiovasc Engineering 2008 8 174 84 10.1007/s10558-008-9054-z 18543102

[B9] Aljuri N Cohen RJ Theoretical considerations in the dynamic closed-loop baroreflex and autoregulatory control of total peripheral resistance Am J Physiol 2004 287 H2252 2273 10.1152/ajpheart.00489.2003 15231503

[B10] Hay I Rich J Ferber P Burkhoff D Maurer MS Role of impaired myocardial relaxation in the production of elevated left ventricular filling pressure Am J Physiol 2005 288 H1203 H1208 10.1152/ajpheart.00681.2004 15498827

[B11] Ramachandran D Luo C Ma TS Clark JW Using a human cardiovascular-respiratory model to characterize cardiac tamponade and pulsus paradoxus Theor Biol Med Model 2009 6 15 10.1186/1742-4682-6-15 19656411 PMC2736922

[B12] Murgo JP Westerhof N Giolma JP Altobelli SA Aortic input impedance in normal man: relationship to pressure waveforms Circulation Res 1980 62 105 16 10.1161/01.cir.62.1.105 7379273

[B13] Murgo JP Westerhof N Input impedance of the pulmonary arterial system in normal man: effects of respiration and comparison to systemic impedance Circulation Res 1984 54 666 73 6733863 10.1161/01.res.54.6.666

[B14] Chung DC Niranjan SC Clark JW Jr Bidani A Johnston WE Zwischenberger JB Traber DL A dynamic model of ventricular interaction and pericardial influence Am J Physiol 1997 272 H2942 2962 9227574 10.1152/ajpheart.1997.272.6.H2942

[B15] Ohno Y Hatabu H Murase K Higashino T Kawamitsu H Watanabe H Takenaka D Fuji M Sugimura K Quantitative assessment of regional pulmonary perfusion in the entire lung using three-dimensional ultrafast dynamic contrast-enhanced magnetic resonance imaging: preliminary experience in 40 subjects J MRI 2004 20 353 365 10.1002/jmri.20137 15332240

[B16] Cash JR Karp AH A variable order Runge-Kutta method for initial value problems with rapidly varying right-hand sides ACM Trans Math Soft 1990 16 201 22 10.1145/79505.79507

[B17] Baicu CF Zile MR Aurigemma GP Gaasch WH Left ventricular systolic performance, function, and contractility in patients with diastolic heart failure Circulation 2005 111 18 2306 12 10.1161/01.CIR.0000164273.57823.26 15851588

[B18] Kass DA Bronzwaer JG Paulus WJ What mechanisms underlie diastolic dysfunction in heart failure? Circ Res 2004 94 12 1533 42 10.1161/01.RES.0000129254.25507.d6 15217918

[B19] Park HS Naik SD Aranow WS Visintainer PF Das M McClung JA Belkin RN Velocity by tissue Doppler imaging in the evaluation of left ventricular diastolic function J Am Coll Cardiol 2006 98 970 2 10.1016/j.amjcard.2006.04.044 16996885

[B20] Shabetai R Fowler NO Fenton JC Masangkay M Pulsus paradoxus Journal of Clinical Investigation 1965 44 11 1882 1898 10.1172/JCI105295 5843718 PMC289688

[B21] Mandinov L Eberli FR Seiler C Hess OM Diastolic heart failure Cardiovasc Res 2000 45 813 25 10.1016/S0008-6363(99)00399-5 10728407

[B22] Zile MR Heart failure with preserved ejection fraction: is this diastolic heart failure? J Am Coll Cardiol 2003 41 9 1519 22 10.1016/S0735-1097(03)00186-4 12742292

